# Highly Conductive Polyoxanorbornene‐Based Polymer Electrolyte for Lithium‐Metal Batteries

**DOI:** 10.1002/advs.202302932

**Published:** 2023-07-17

**Authors:** So Young An, Xinsheng Wu, Yuqi Zhao, Tong Liu, Rongguan Yin, Jung Hyun Ahn, Lynn M. Walker, Jay F. Whitacre, Krzysztof Matyjaszewski

**Affiliations:** ^1^ Department of Chemistry Carnegie Mellon University 4400 Fifth Avenue Pittsburgh PA 15213 USA; ^2^ Department of Materials Science and Engineering Carnegie Mellon University 5000 Forbes Avenue Pittsburgh PA 15213 USA; ^3^ Department of Chemical Engineering Carnegie Mellon University 5000 Forbes Avenue Pittsburgh PA 15213 USA; ^4^ Scott Institute for Energy Innovation Carnegie Mellon University 5000 Forbes Avenue Pittsburgh PA 15213 USA

**Keywords:** batteries, high conductivity, polymeric electrolytes, ROMP, Solid polymer electrolytes

## Abstract

This present study illustrates the synthesis and preparation of polyoxanorbornene‐based bottlebrush polymers with poly(ethylene oxide) (PEO) side chains by ring‐opening metathesis polymerization for solid polymer electrolytes (SPE). In addition to the conductive PEO side chains, the polyoxanorbornene backbones may act as another ion conductor to further promote Li‐ion movement within the SPE matrix. These results suggest that these bottlebrush polymer electrolytes provide impressively high ionic conductivity of 7.12 × 10^−4^ S cm^−1^ at room temperature and excellent electrochemical performance, including high‐rate capabilities and cycling stability when paired with a Li metal anode and a LiFePO_4_ cathode. The new design paradigm, which has dual ionic conductive pathways, provides an unexplored avenue for inventing new SPEs and emphasizes the importance of molecular engineering to develop highly stable and conductive polymer electrolytes for lithium‐metal batteries (LMB).

## Graphical abstract

Polyoxanorbornene‐based bottlebrush polymers with PEO side chains for solid polymer electrolytes (SPE) are presented. This SPE demonstrates high ionic conductivity (7.12 × 10^−4^ S cm^−1^) and excellent electrochemical performance when combined with Li metal anodes and LiFePO_4_ cathodes. This innovative design with dual ion‐conducting pathways offers new possibilities for stable and conductive polymer electrolytes in lithium‐metal batteries.

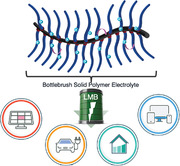

## 1 Introduction

An economically viable and high‐performing energy storage solution has been a long‐term challenge for accommodating the fast‐growing global energy rate demand. As society moves away from using the internal combustion engine (ICE), increasing attention is being paid to converting renewable energy sources to electrical energy that can be stored in an efficient energy storage system.^[^ ^1^, ^2^ ^]^ Rechargeable batteries, such as Lithium‐Ion Batteries (LIBs), are excellent sustainable energy storage solutions since they generate far fewer greenhouse gases than the ICE and still provide relatively high energy density and power. However, a state‐of‐the‐art LIB configuration provides only a cell‐level energy density of ≈ 250 Wh kg^−1^, far less than the ICE, which limits the practical usage of LIBs in advanced applications such as electric vehicles and grid storage.^[^ ^3^, ^4^ ^]^ Although recent advances and extensive research in LIB technology—including organic electrodes,^[^ ^5^, ^6^, ^7^, ^8^, ^9^, ^10^, ^11^, ^12^, ^13^, ^14^, ^15^ ^]^ anode engineering,^[^ ^16^, ^17^, ^18^, ^19^, ^20^, ^21^, ^22^, ^23^ ^]^ and new classes of layered metal oxide cathodes^[^ ^24^, ^25^, ^26^, ^27^, ^28^ ^]^—have demonstrated promising performance, the energy density of LIBs continues to be a bottleneck.

Rechargeable batteries that use metallic Li as anodes are considered to be one of the most promising solutions for achieving high energy density at the full cell level. Lithium metal has the lowest redox potential (−3.04 V vs the standard hydrogen electrode) and a high theoretical capacity (3860 mA h g^−1^) that is far superior to the commercialized graphite anode (375 mA h g^−1^).^[^ ^29^, ^30^ ^]^ Unfortunately, LMBs are commonly highly reactive to moisture and elevated temperatures, thus exposing them to safety concerns, including fires and explosions. In particular, the conventional liquid electrolytes in current LIBs are incompatible with LMBs due to the harmful growth of lithium dendrites that can cause complete battery failure.^[^ ^17^, ^31^, ^32^ ^]^ Certainly, overcoming these challenges by modifying the current design of LIBs should be a crucial research focus for developing safe and high‐performance LMBs.

An SPE can overcome these drawbacks—and the safety concerns of the liquid electrolytes in LMBs—due to its non‐flammable nature, a wide range of operating temperatures (−30 to 100 °C), and safe/stable contact with Li metal.^[^ ^33^, ^34^, ^35^, ^36^, ^37^, ^38^ ^]^ Various functional polymers have been studied as SPEs, including polymer/ceramic composites,^[^ ^31^, ^39^ ^]^ polycarbonate‐,^[^ ^40^, ^41^, ^42^ ^]^ polyester‐,^[^ ^43^, ^44^, ^45^ ^]^ and poly(ethylene oxide) (PEO)‐based polymers.^[^ ^31^, ^33^, ^46^, ^47^ ^]^ Among these candidates, PEO has been the most investigated polymer since it possesses multiple oxygen atoms that can coordinate with Li‐ions, thus efficiently promoting the ion conduction pathway within the matrix. However, PEO has high crystallinity at room temperature that interferes with polymer segmental motion and Li‐ion movement. As a result, sluggish Li‐ion kinetics ultimately lead to the poor conductivity (≈10^−8^ to 10^−5^ S cm^−1^ at room temperature) of PEO‐based polymer electrolytes at optimal LMB operation temperature, which hampers overall battery performance, including poor rate capability, cycling stability, and low energy/power.^[^ ^30^, ^33^, ^37^ ^]^ In addition, the poor mechanical strength of PEO in solid‐state cannot physically prevent the dangerous penetration of lithium dendrites at high temperatures or high current density, which is a desired metric for the industrial application/commercialization of LMBs.

To circumvent these issues, various composite electrolytes have been explored to decrease the crystallinity of PEO so that both ionic conductivity and mechanical strength are enhanced. Incorporating inorganic parts, including garnet‐type, perovskite, and/or sodium (NA) super ionic conductor (NASICON), is an effective strategy to enhance the polymer segmental motions and provide additional Li^+^ transport channels.^[^ ^48^, ^49^, ^50^ ^]^ However, these inorganic additives often need better processibility, are incompatible with electrodes with high interfacial resistance, and are made from expensive raw materials compared to polymer electrolytes. Aside from these composite solid electrolytes, many creative polymeric design strategies have been reported to finely tune the morphology and architecture of the polymers to enhance their mechanical strength and ionic conductivity without any help from inorganic fillers or plasticizers. Controlled polymerization techniques, including atom transfer radical polymerization (ATRP), reversible addition‐fragmentation chain‐transfer polymerization (RAFT), and ring‐opening metathesis polymerization (ROMP), have been actively employed to construct a higher‐ordered nanostructure in SPEs through the block, graft, hyperbranched, and star co‐polymers.^[^ ^51^, ^52^, ^53^, ^54^, ^55^, ^56^, ^57^, ^58^, ^59^, ^60^, ^61^ ^]^ For example, triblock copolymers such as polystyrene (PS)‐*b*‐PEO‐*b*‐polystyrene (PS) and PS‐*b*‐poly(ethylene glycol) methacrylate (PEOMA)‐*b*‐PS have been studied as SPEs.^[^ ^54^, ^62^, ^63^ ^]^ Such designs can provide efficient ionic conductive pathways through ethylene oxide (EO) repeat units from PEO or PEOMA, and a mechanical strength contributed by PS blocks. However, these structures consist of inevitable non‐conductive areas at the interface of PEO/PS or within PS blocks where the Li‐ion transport is rather limited (ionic conductivity, σ ≈10^−6^–10^−5^ at RT). Others have demonstrated that bottlebrush polymers with denser PEO sidechains can promote interchain hopping of Li‐ions, thereby facilitating the transportation of the ions.^[^ ^61^, ^63^, ^64^, ^65^, ^66^ ^]^ Further, these bottlebrush polymer electrolytes have exhibited ideal thermal and mechanical properties. Recently, a series of well‐defined bottlebrush‐like polymers with densely grafted PEO side chains through ATRP and RAFT was reported to prepare solid polymer electrolytes.^[^ ^67^ ^]^ These SPEs showed a moderate ion conductivity of 2.51×10 ^−5^ S cm^−1^ at 30 °C, great rate capability (157 mA h g^−1^ at 0.1 C and 122.7 mA h g^−1^ at 0.5 C with LiFePO_4_ cathode and Li metal anode), and cycling stability (98% capacity retention over 100 cycles) at the elevated temperature (60 °C). Although these higher‐ordered architectures have greatly increased the practical usage of the polymer electrolytes for LMB, their low room temperature ionic conductivity remains a significant obstacle impeding the overall battery performance, including practical capacity, rate capability, cycling stability, and operating voltage. Additionally, the ionic conductive groups are limited to grafted side chains of these block copolymer or bottlebrush polymers, and thus the importance of continuous ion conduction pathways on the polymer's backbone often is overlooked. Therefore, it is of interest to explore the synthesis of polymers that possess multiple Li‐ion coordination sites both at the side chains and the polymer backbones, which leads to the practical conductivity target of σ > 4×10^−4^ S cm^−1^ at room temperature for SPEs.^[^ ^52^ ^]^

**Figure 1:**
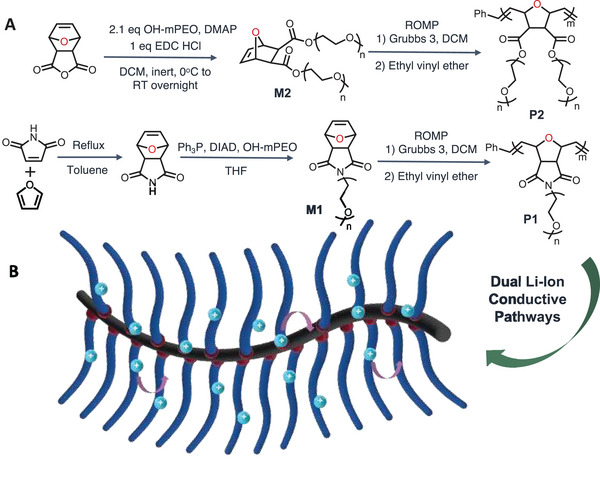
Preparation of polymers. A) Synthesis of bottlebrush polymer P2 and P1. B) Schematic of bottlebrush polymer electrolytes, illustrating the dual Li‐ion movements.

Here, we describe bottlebrush polymer‐based solid electrolytes with dual ionic conducting groups at the polymeric backbones and the pendant groups (**Figure** **1**A,B). Polyoxanorbornene derivatives with oxygen atoms in the polymer's backbone can increase the rate of Li‐ion transport.^[^ ^68^, ^69^, ^70^, ^71^ ^]^ Moreover, traditional pendant ethylene oxide (EO) repeat units provide additional ionic conduction pathways and a lower crystallinity than the long EOs of linear polymers. Many recent studies have introduced complicated architectures and multiple synthetic steps to install functional groups to facilitate Li‐ion transport within SPEs. Interestingly, the present study provides a practical and simple design strategy for introducing an ionic conducting group on the polymer backbone by tweaking monomer structures to significantly enhance ionic conductivity at room temperature. Thus, we explored two oxanorbornene‐based polymers (P1 and P2) and presented their stability/electrochemical performance. P2 (di‐functionalized PEO, poly(ethylene oxide), side chains) with ester linkages ensure the installation of a maximum number of PEO pendant units, whereas P1 (mono‐functionalized PEO side chain), comprised of the less hindered EO repeat units through ether linkages, can increase the electrochemical stability of the material under negative redox potential. This unique bottlebrush polymer design for SPEs greatly boosts Li‐ion transport in the solid‐state, which leads to outstanding battery performance comparable to traditional liquid electrolyte systems.

## 2 Results and Discussion

### 2.1 Synthesis and Characterization of Macromonomers (M1 and M2) and Bottlebrush Polymers (P1 and P2)

We designed polymers to incorporate the EO repeat units into an oxanorbornene backbone. First, we prepared the M2 macromonomer using the esterification of the pendant group with an oxanorbornene backbone. Note that the series of M2 was prepared with different molar mass (*M*_n ave_ = 912, 2210, and 4060 g mol^−1^) by varying the molar mass of monomethoxy‐terminated PEO (OH‐mPEO, *M*_n__ave_ = 350, 1000, and 2000 g mol^−1^) (Table S1, Supporting Information). Then, we synthesized the oxanorbornene precursor with imide linkage through the Diels–Alder reaction, following a previously reported protocol.^[^ ^72^ ^]^ Subsequently, we synthesized M1 with an OH‐mPEO chain (*M*_n ave_ = 1000 g mol^−1^) by a Mitsunobu‐type coupling reaction with 84% yield. We polymerized these M1 and M2 macromonomers (note that M1 contains 1 PEO and M2 contains 2 PEO chains) using the ROMP method with a Grubbs third‐generation catalyst (G3)—which is well‐known to successfully polymerize norbornenes with sterically hindered or bulky substituents —to prepare the bottlebrush‐type polymers (P1 and P2). We conducted the polymerization using different monomer‐to‐catalyst ratios to target a specific degree of polymerization (DP) using monomers at varied concentrations of 0.14–0.04 m in dichloromethane. Detailed ROMP conditions for each M2 and M1 macromonomer and P2 and P1 characterizations are listed in **Tables** **1** and **2**. Polymer electrolytes typically require a higher molecular mass (> 150 kg mol^−1^) to gain good mechanical integrity while retaining flexibility. Thus, we performed a series of polymerization by varying the ratio of G3 to the macromonomers in an attempt to prepare polymers with a higher DP of bottlebrush backbone. For convenience, the DP of the bottlebrush backbone is denoted as DP_bb,_ and the DP of the side chain, EO repeat units, is denoted as DP_sc_. First, we studied the ROMP of the M2 macromonomer with two PEO chains. A complete macromonomer consumption was obtained when the molar mass of M2 was relatively lower (i.e., M_n ave_ = 912 g mol^−1^) with low DP_bb_ (< 50). This result was confirmed using ^1^H‐NMR, by which we monitored the disappearance of alkene protons at 6.43 ppm for M2 macromonomers. Additionally, we observed a new broad alkene peak from 5.94 to 5.57 ppm representing the formation of P2 (Figures S1 and S3, Supporting Information). However, the efficiency of the ROMP process of M2 was affected by the molecular weight of the macromonomers. For example, a further increase in M2 molecular mass (up to 4059 g mol^−1^) with DP_bb_ (up to 50) resulted in incomplete monomer conversion or a broader molecular weight distribution/multimodal distribution in gel permeation chromatography (GPC) traces (Figures S16 and S17, Supporting Information). The lower effectiveness of the ROMP of M2 is attributable to the sterically hindered di‐functionalized macromonomers where the propagating polymer chain ends have reduced reactivity with macromonomers as the DP_bb_ of polymerization increases.^[^ ^73^ ^]^ On the other hand, the polymerization of the M1 macromonomer was much more straightforward owing to its less crowded mono‐substituted PEO chains. P1 can be polymerized up to DP_bb_ 300 and have symmetrical GPC traces with a narrow molecular distribution (*Đ* < 1.2) for all series of P1 (from DP_bb_ 50 to DP_bb_ 300; Figure S18, Supporting Information). For example, P1 with a macromonomer M1 (*M*_n ave_ = 1147 g mol^−1^)‐to‐catalyst ratio of 150:1 showed improved control over the polymerization (*M*_n theo_ = 172 kg mol^−1^, M_n abs_ = 191 kg mol^−1^, *Đ* = 1.15). We calculated the conversion of the M1 macromonomer similar to the conversion of M2. Specifically, we monitored the disappearance of the vinyl proton peaks of M1 at 6.51 ppm (Figure S6, Supporting Information) and the appearance of the polymer proton peaks of the P1 bottlebrush polymers at 6.12–5.78 ppm (Figure S8, Supporting Information). Next, we carried out a thermogravimetric analysis to study the thermal stability of the P1 and P2 polymers and the respective M1 and M2 macromonomers (Figures S19 and S20, Supporting Information). The good thermal stability (>200 °C) of SPEs also is a practical consideration due to the rising safety issues around lithium‐ion batteries and the wide operating conditions required for LMBs. Compared to macromonomers (M1 and M2), their polymer counterparts (P1 and P2) showed increased thermal stability with slightly higher decomposition temperatures.

**Table 1:** Results of ROMP of M2 with a different molecular mass of macromonomer

Entry	[G3]:[M]	M2_ave_ [g mol^−1^]	*M*_n_, _theo_ [kg mol^−1^]	DP_bb_	DP_sc_	Conv [%]	*M*_n_, _GPC_ [kg mol^−1^]	*Đ*
P2‐1A	1:10	912	9.12	10	8	≈100	10.7	1.28
P2‐1B	1:25	912	21.8	25	8	≈100	22.9	1.27
P2‐1C	1:50	912	45.6	50	8	≈100	25.6	1.33
P2‐2A	1:10	2210	22.1	10	23	≈100	16.2	1.29
P2‐2B	1:25	2210	55.3	25	23	88	9.68	1.45
P2‐2C	1:50	2210	111	50	23	50	3.35	2.82
P2‐3A	1:10	4060	40.6	10	45	≈100	1.59	3.41

Reaction conditions: [M2] = 0.14‐0.05 m (5‐9 wt% of macromonomer in the reactive mixture) in dichloromethane at Reaction conditions: [M2] = 0.14–0.05 m (5–9 wt% of macromonomer in the reactive mixture) in dichloromethane at RT stirring 5000 rpm for 2 or 18 h. G3: Grubbs third‐generation catalyst; M2: macromonomer 2; Monomer conversion was determined using ^1^H‐NMR spectroscopy. All measurements were analyzed using GPC (dimethylformamide as eluent) calibrated to poly(methyl methacrylate) standard. M_n_, _theo_: Theoretical number average molar mass; DP_bb_: Degree of polymerization of bottlebrush backbone; DP_sc_: Degree of polymerization of side chain.

**Table 2:** Results of ROMP of M1

Entry	[G3]:[M]	M2_ave_ [g mol^−1^]	*M*_n_, _theo_ [kg mol^−1^]	DP_bb_	DP_sc_	Conv [%]	*M*_n_, _MALS, GPC_ [kg mol^−1^]	*Đ*
P1‐1	1:50	1150	57.4	50	23	≈100	47.0	1.01
P1‐2	1:100	1150	115	100	23	≈100	101	1.14
P1‐3	1:150	1150	172	150	23	≈100	191	1.15
P1‐4	1:300	1150	344	300	23	≈100	304	1.15

Reaction conditions: [M1] = 0.04 m (4.9 wt% of macromonomer in the reactive mixture), in dichloromethane at RT stirring 5000 rpm for 5 h. G3: Grubbs third‐generation catalyst; M1: macromonomer 1; Monomer conversion was determined using ^1^H‐NMR spectroscopy. All measurements were analyzed using GPC (dimethylformamide as eluent). DP_bb_: Degree of polymerization of bottlebrush backbone; DP_sc_: Degree of polymerization of side chain.

### 2.2 Preparation of P1 and P2 Solid Polymer Electrolytes

An ideal polymer electrolyte should have a low glass transition temperature (*T*_g_) at ambient temperature to accelerate the segmental motions within the conductive polymer chains with high ionic conductivity. PEO‐based SPEs such as P1 and P2 can be advantageous due to their intrinsically low *T*_g_ of PEO units. The P1 and P2 with DP_sc_ = 23 show melting transition (*T*_m_) peaks at 35–39 °C due to the semicrystalline nature coming from the longer PEO side chains, while P2 with DP_sc_ = 8 only exhibits a *T*_g_ with no *T*_m_ (Figures S25 and S27, Supporting Information). However, the melting transition associated with PEO chains nearly disappeared upon the addition of LiTFSI salts, indicating that the crystallinity of a long PEO chain can be suppressed by effective interactions between Li^+^ and EO units (Figures S26 and S28, Supporting Information).

**Figure 2:**
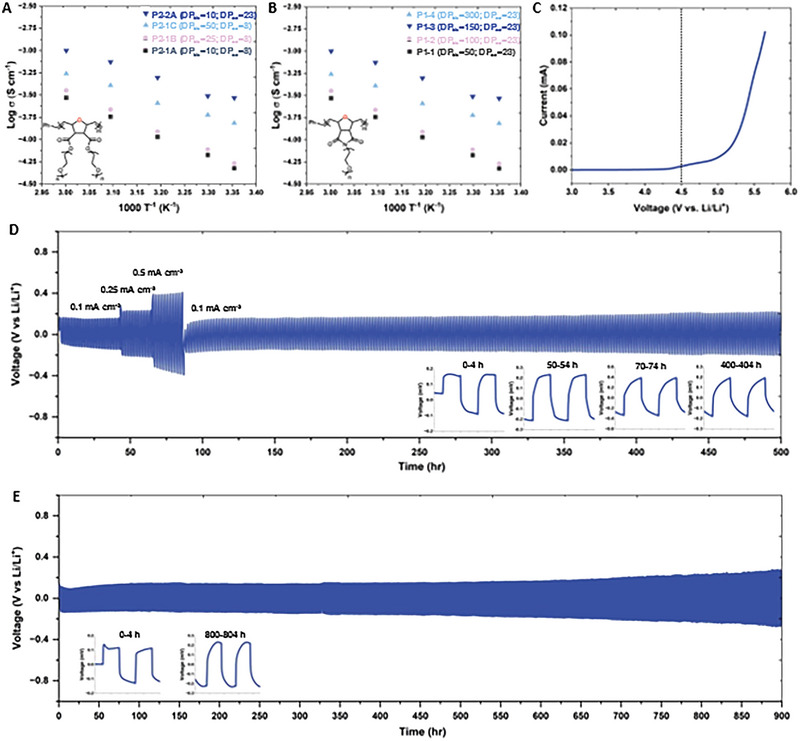
Characterizations of polymer electrolytes. A) Ionic conductivity of P2. B) Ionic conductivity of P1. C) LSV curves of P1. D) Galvanostatic cycling of Li|P1 electrolyte|Li symmetric cell at ambient conditions and different current densities (0.1, 0.25, and 0.5 mA cm^−2^; 0.1, 0.25, and 0.5 mA h cm^−2^). E) Galvanostatic cycling of Li|P1 electrolyte|Li symmetric cell at a constant current density of 0.1 mA cm^−2;^ 0.1 mA h cm^−2^ over 900 h at room temperature.

**Figure 3:**
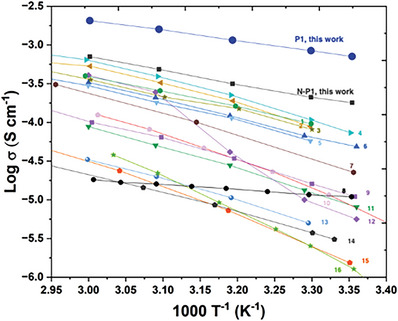
Comparison of the polyoxanorbornene (P1) polymer electrolyte to other polymer electrolyte systems. 1,^[^ ^76^ ^]^ 2,^[^ ^67^ ^]^ 3,^[^ ^77^ ^]^ 4,^[^ ^78^ ^]^ 5,^[^ ^79^ ^]^ 6,^[^ ^66^ ^]^ 7,^[^ ^80^ ^]^ 8,^[^ ^81^ ^]^ 9,^[^ ^82^ ^]^ 10,^[^ ^83^ ^]^ 11,^[^ ^66^ ^]^ 12,^[^ ^66^ ^]^ 13,^[^ ^84^ ^]^ 14,^[^ ^85^ ^]^ 15,^[^ ^60^ ^]^ 16.^[^ ^69^ ^]^ The detailed structure and ionic conductivity at ambient conditions are summarized in Table S3 (Supporting Information).

Previous studies have suggested that polymer‐brush electrolytes with PEO side chains decreased ionic conductivity as the side chain length was shortened.^[^ ^66^, ^74^ ^]^ These studies claimed that such behavior was due to the low segmental mobility of PEO chains near the backbone and that this low utility of PEO units can be detrimental to overall ionic conductivity. Thus, more EO repeat units can further enhance the conductivity because the percentage of non‐conductive EO units versus total EO units has decreased. In addition, the central EO unit along the side PEO chains has a better probability of coordinating with the Li‐ions than the EO units close to the backbone and side chain tails. However, a competitive effect exists due to the semi‐crystalline nature of PEO for longer chains. A longer PEO displays heterogeneous conducting environments (even after the addition of Li salts) in which crystallized regions provide very limited ion motions.^[^ ^66^, ^75^ ^]^ P1 and P2‐based polymer electrolytes follow a similar trend (**Figure** **2**A,B). Note that we calculated ionic conductivity by Equation S1 (Supporting Information) using electrochemical impedance spectroscopy (EIS, see the corresponding values in **Table** **3**). We found that the ionic conductivity of the P2‐based polymer electrolytes increased with a higher DP_bb_ of polymers (longer polyoxanorbornene backbone) or a higher macromonomer molecular weight (more EO repeat units in the side chains; higher DP_sc_). P1‐based polymer electrolytes showed a more substantial increase in ionic conductivity as the DP_bb_ of the polymer increased from 50 to 150. It is worth noting that our P1 polymer with DP_bb_ = 150 presented the ionic conductivity of 7.12 × 10^−4^ at room temperature and 2.05 × 10^−3^ S cm^−1^ at 60 °C. To the best of our knowledge, such a high room temperature conductivity has never been reported in a solid polymer electrolyte system, which can directly affect the overall performance of LMB when paired with P1 electrolytes (**Figure** **3** and Table S3, Supporting Information). We reason that the high ionic conductivity of P1 is attributable to our design strategies with dual conductive pathways within bottlebrush polymers, by which extremely effective ionic conduction is possible from both the backbone and side chains of the polymers. However, the overall ionic conductivity of the P1 electrolyte reached its maximum at DP = 150 and then started to decrease, as seen in P1‐4 (Table 3; P1 with DP_bb_ = 300). Specifically, the ionic conductivity of P1 electrolytes decreased to 6.92×10^−4^ at RT and 1.97×10^−3^ S cm^−1^ at 60 °C as the DP_bb_ of the polymer increased from 150 to 300. The longer bottlebrush P1 can benefit SPEs by efficiently promoting ion conduction within the side chain through interchain hopping and providing relatively good mechanical properties. Also, these bottlebrush P1 polymers with high DP_bb_ can stimulate Li‐ion transport via the backbone through inter‐ and intra‐chain hopping mechanisms. In contrast, the polymer backbone becomes more rigid with a higher DP_bb_ (i.e., DP_bb_ = 300), and thus, after a certain point, it can limit ion conduction at the oxanorbornene polymer backbones, which results in a lower ionic conductivity value. We also conducted a few control experiments to determine the ionic conductivities of the M1 macromonomer and the norbornene‐based P1 (N‐P1) (Figure S21 and Table S2, Supporting Information). A detailed synthesis and preparation of the N‐P1 are presented in Figure S9 (Supporting Information). N‐P1 polymer electrolyte showed much lower ionic conductivity (1.80×10^−4^ S cm^−1^ at RT) compared to any oxanorbornene‐based P1 polymer electrolyte presented in this study. M1 macromonomer offered a slightly higher RT ionic conductivity (8.24×10^−4^ S cm^−1^) than the P1 with DP_bb_ 150. The lower ionic conductivity of the bottlebrush polymer compared to its corresponding non‐polymerized free PEO macromonomer is a common feature because these flexible macromonomers have fewer hindered solvation sites and better chain mobility.^[^ ^66^ ^]^ However, the low mechanical properties of M1 compared to P1 are a serious drawback of SPEs, which cannot efficiently suppress dendrite growth with low cycling stability. Based on this extensive screening, we learned that the polymerization of oxanorbornene‐derived M1 was an absolute requirement for preparing functional SPEs for the lithium metal anode. For all the remaining electrochemical studies, we chose and focused on P1 electrolytes with DP_bb_ = 150 and DP_sc_ = 23, which provided the highest ionic conductivity of the prepared polymer series.

**Table 3:** Summary of ionic conductivities for P1 and P2

Entry	M1 or M2 [g mol^−1^]	DP_bb_	DP_sc_	σ [S cm^−1^] at RT	σ [S cm^−1^] at 60 ^o^C
P2‐1A	912	10	8	4.75 × 10^−5^	2.95 × 10^−4^
P2‐1B	912	25	8	5.38 × 10^−5^	3.56 × 10^−4^
P2‐1C	912	50	8	1.53 × 10^−4^	5.49 × 10^−4^
P2‐2A	2210	25	23	2.93 × 10^−4^	9.99 × 10^−4^
P1‐1	1150	50	23	2.19 × 10^−4^	1.00 × 10^−3^
P1‐2	1150	100	23	3.31 × 10^−4^	1.15 × 10^−3^
P1‐3	1150	150	23	7.12 × 10^−4^	2.05 × 10^−3^
P1‐4	1150	300	23	6.92 × 10^−4^	1.97 × 10^−3^

**Figure 4:**
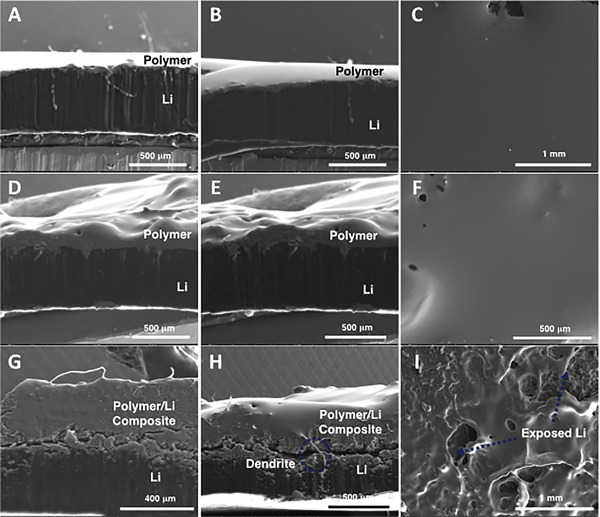
SEM images of cycled lithium electrodes. A,B) Cross‐sectional views of Li electrode with a P1 polymer layer before cycling. C) Top view of Li electrode with a P1 polymer layer before cycling. D,E) Cross‐sectional views of Li electrode with a P1 polymer layer after 400 h. F) Top view of Li electrode with a polymer layer after 400 h. G,H) Cross‐sectional views of Li electrode with a P1 polymer layer after 800 h. I) Top view of Li electrode with a P1 polymer layer after 800 h.

One of the main advantages of SPEs is their excellent stability over a wide range of voltage, thus creating numerous possibilities to be paired with different cathodes, including Lithium Iron Phosphate (LFP), Lithium Cobalt Oxide (LCO), and high‐voltage Lithium Nickel Cobalt Manganese (NCM). Figure 2C demonstrates the outstanding electrochemical stability of the P1 electrolyte from 3.0 V up to 4.5 versus Li^+^/Li, confirmed by linear sweep voltammetry (LSV). We then employed the symmetric cell structure, Li|P1 electrolyte|Li, to perform constant current polarization tests over extensive cycles at different current densities (Figure 2D). Each charge and discharge cycle was set to be 1 h at the current density of 0.1, 0.25, 0.5, and then back to 0.1 mA cm^−2^ at RT. During the repeated cycles, the Li‐ions were plating/stripping the Li metal electrodes to mimic the charging and discharging operation. The cells with P1 electrolyte had a stable, low overpotential plateau of 0.14. 0.22, 0.38 V at 0.1. 0.25 and 0.5 mA cm^−2^, respectively. Then, the overpotential fell back to 0.14 V when the current density decreased to 0.1 mA cm^−2^, but slowly increased to 0.19 V upon 500 h of cycling. The same symmetric cell structure with P1 electrolytes was tested for long‐term cyclability at 0.1 mA cm^−2^ for 900 h (Figure 2E). The cell exhibited stable symmetric voltage profiles of −0.15 to 0.15 V up to 200 h and then slightly increased to −0.19 to 0.19 V at 400 h for 0.1 mA cm^−2^ current density, followed by a further increase in overpotential to −0.22 to 0.22 V at 800 hrs. As a comparison, we assembled and tested the same symmetric cell structure using the M1 macromonomer as an electrolyte, which showed similarly high ionic conductivity comparable to the P1 polymers (Figures S21 and S22, Supporting Information). During constant charge/discharge, the Li|M1 electrolyte|Li performed at a much higher asymmetrical overpotential from −0.39 to 0.54 V at a maximum compared to the P1 electrolyte. This highly polarized voltage profile was indicative of unwanted build‐up of solid electrolyte interphase (SEI) and uneven lithium deposition over the cycles. These results suggested that the stability of the P1 polymer electrolytes is better at suppressing the Li dendrite formation than M1. In addition, the morphology of Li electrodes after galvanostatic polarization was studied to evaluate the lithium deposition during cycling by Scanning Electron Microscopy (SEM). (**Figure** **4**). The cycled Li metal electrode, after 400 h, maintained a smooth polymer layer and good contact with lithium, suggesting no significant dendrite formation. On the other hand, the polymer layer became much more heterogenous and formed an additional polymer/Li composite upon severe cycling (up to 800 h). Upon prolonged cycling, the interphase between the Li electrode and polymer or polymer/Li composite started to become unstable with visible cracks, thus exposing the Li layer, and small dendrites’ growth was observed throughout the layers. These post‐mortem SEM studies suggest that our P1 electrolyte can potentially withstand severe cycling by successfully slowing down the dendritic formation and providing good interfacial compatibility with the Li metal electrode. While the P1 electrolyte exhibits the characteristics of a viscoelastic solid with a relatively low shear modulus (Figure S32, Supporting Information), it has demonstrated remarkable dendrite resistance for over 800 h of stripping/plating cycles.

### 2.3 Electrochemical Characterization of Solid Polymer Electrolytes

**Figure 5:**
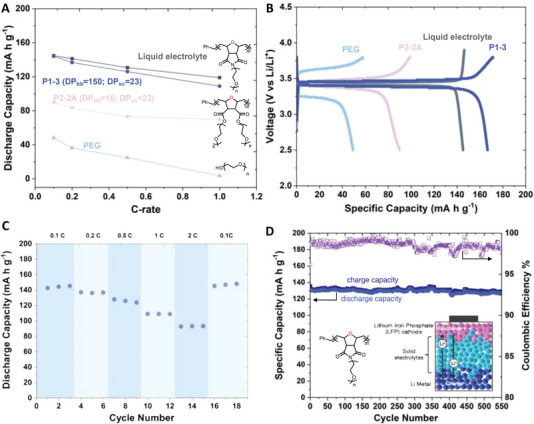
Electrochemical performance of P1 and P2 electrolytes with LFP cathode at room temperature. A) Discharge capacity retention versus C‐rate. B) Charge/discharge profiles of P1, P2, liquid electrolyte, and PEO at 0.1 C. C) The rate capability test of P1. D) Cycling stability of P1 at 0.5 C. Note that the theoretical capacity of LFP was set to be 150 mA h g^−1^ for all testing to calculate the C‐rates.

To test the P1 electrolytes’ feasibility with a commercially available cathode, we fabricated a model solid‐state battery—a Li|P1 electrolyte|LiFePO_4_ (LFP) cell—and conducted potential‐limited galvanostatic charge and discharge cycling (GCD) experiments between the charge and discharge potential limits from 3.8 to 2.5 V (**Figure** **5**). Prior to the studies, the electrochemical stability of P1 electrolytes was evaluated when paired with the LFP cathode. Cyclic voltammetry of Li|P1 electrolyte|LFP cells have one reversible peak centered at 3.43 V, corresponding to the chemical oxidation/delithiation of LFP active materials (Figure S30, Supporting Information). Next, the assembled half‐cell was charged to 3.2 V and held at a gradually high voltage for 20 h up to 3.8 V (Figure S31, Supporting Information). Within the operating voltage limit for LFP, the current was stabilized with a plateau in each voltage limit. These results together suggest that our P1 polymer is electrochemically stable and can be safely paired with an LFP cathode. Next, we investigated the rate capabilities of the LFP cathode with our P1 electrolytes at various current densities of 0.1 to 2 C‐rate. We used an asymmetric charging/discharging protocol as the means to determine the feasibility of SPEs in LMB, and it enabled us to conduct non‐biased rate performance studies for the SPE system, which fully utilized the active materials of the cathodes. Figure 5B shows the typical stable charge/discharge plateaus at 3.47 V and 3.40 V, which demonstrates a reversible electrochemical reaction. The discharge capacities of P1 during the rate capability test gradually decreased with an increase in current densities (Figure 5C). For example, Li|P1|LFP delivered discharge capacities of 144.1, 136.8, 124.0, 109.0, and 93.0 mA h g^−1^ at 0.1, 0.2, 0.5, 1, and 2 C‐rate. More importantly, the LFP half‐cell with a P1 electrolyte was able to deliver full initial capacity (145.3 mA h g^−1^), which is close to 100% of the theoretical capacity of LFP. To highlight the adaptability of P1 in LMB, we compared its discharge capacity in the first cycle (after formation cycles) with other batteries assembled as Li|P2 electrolyte (with P2‐2A polymer) |LFP and Li|liquid electrolyte (1 m Lithium hexafluorophosphate 1:1:1(v/v/v) = ethylene carbonate: dimethyl carbonate: diethyl carbonate) |LFP and lastly Li|PEO_1000_ electrolyte|LFP after the formation cycles (Figure 5A,B). Remarkably, Li|P1|LMB showed very similar discharge capacities to the Li|Liquid electrolyte|LFP control sample, whereas the Li/P2/LFP, with a much lower ionic conductivity, could only deliver approximately half of its theoretical capacity, even at the initial cycle with low C‐rates. In addition, Li|PEO|LFP exhibited the lowest ionic conductivity with highly polarized charge/discharge curves during its rate capability test. These results were expected for P2 and PEO with low room temperature (RT) ionic conductivity, as observed in many SPEs. The poor conductivities of these SPEs often require enhancement by increasing the battery operation temperature (> 40 °C) or making composite electrolytes by incorporating non‐conductive inorganic fillers^[^ ^48^, ^86^ ^]^ or conductive garnet‐type fillers.^[^ ^31^, ^87^, ^88^ ^]^ In contrast, the highly conductive P1 electrolyte has demonstrated excellent compatibility with the commercialized LFP cathode at room temperature without modifying the polymer electrolyte into composite materials or other battery operation conditions.

To gain more insights into the stability of Li|P1 electrolyte|LFP cells, we studied their long‐term cycling stability over 550 cycles between the charge and discharge potential limits of 3.8 and 2.5 V (Figure 5D). We performed these cycling tests by symmetric charging/discharging at the same current densities. The cell with the P1 electrolyte reached stable discharge capacities of 132.1 mAh g^−1^ after three cycles at 0.5 C. The discharge capacities were 132.4 mA h g^−1^ at 50 cycles and 131.7 mA h g^−1^ at 100 cycles (Figure S23, Supporting Information). Overall, the capacity remained at 127.4 mA h g^−1^ even after 550 cycles, with a capacity retention of 97%. The Coulombic efficiency (CE) of the battery during cycling increased in the first initial cycles (from 96% to 99%) due to the gradual formation of stable solid electrolyte interphase coatings on the surface of the lithium electrodes, which can be seen from the cross‐section SEM in Figure 4D,E and confirmed a good contact with the P1 electrolyte and Li. During the cycling, the CE reached over 97% and remained at this high level for over 550 cycles, which implied great reversibility of the LFP materials paired with the P1 electrolyte. The good cyclability and superior rate capability of P1 can be ascribed to our bottlebrush designs with the great binding and transport ability of Li‐ions with oxanorbornene backbones, which leads to the excellent conductivity of the P1 electrolyte. In addition, the high molecular weight of P1 also contributes to long‐term stability with limited dendrite growth by providing the required mechanical strength and good contact with electrodes.

## 3 Conclusion

In summary, we have demonstrated that polyoxanorbornene with pendant PEO side chains is an appropriate candidate for use in SPEs. The unique dual conductive pathways of P1 and P2, through the backbone and side chains of the polymer, greatly increase the ionic conductivity of SPEs. Our careful screening of these oxanorbornene‐based polymers prepared by ROMP suggests that the P1 with DP 150 provides the most optimal mechanical strength and the highest ionic conductivity (7.12 × 10^−4^ S cm^−1^ room temperature) of all the polymer P1 and P2 series. P1 SPE showed excellent voltage stability and stable contact with Li metal during long‐term symmetric cycling. Remarkably, when we assembled our model lithium‐metal batteries with our P1 electrolyte (Li|P1 electrolyte|LFP), these batteries presented superior electrochemical performance, such as strong performance at intermediate high specific currents (144.1 and 93.0 mA h g^−1^ at 0.1 and 2 C‐rate) and cycling stability (97% of capacity retention over 550 cycles. We designed highly successful polymer electrolytes with good/tunable mechanical integrity as well as a high Li‐ion conductivity for all‐solid‐sate batteries with metallic Li anodes. The simplicity of our molecular design with additional oxygen atoms on the polymer backbones enabled us to exceed the state‐of‐the‐art values for the ionic conductivity of SPEs, thus opening numerous possibilities and success for creating safer LMBs when paired with SPEs.

## 4 Experimental Section

### Materials and Instrumentation

All reagents were purchased from Sigma–Aldrich and used as received. ^1^H‐NMR and ^13^C‐NMR spectra were recorded using a Bruker Advance III 500 MHz. Chemical shifts are reported in ppm at room temperature using the solvent peaks of CDCl_3_ at 7.26 ppm and DMSO‐d_6_ at 2.50 ppm. Gel permeation chromatography (GPC) measurements of polymers were performed using PSS columns with DMF as eluent at 50 °C and the flow rate of 1 mL min^−1^. Linear poly(methyl methacrylate) standard were used for calibration. Thermogravimetric Analysis (TGA) was performed on TA instruments 2950 under a nitrogen atmosphere with a flow rate of 60 mL min^−1^. The polymers' glass transition temperatures (*T*_g_) were measured by Differential Scanning Calorimetry (DSC) with TA Instrument QA‐2000 with a heating/cooling rate of 10 °C min^−1^. Scanning Electron Microscope (SEM) was performed for cathode surface morphology investigation and conducted on a Quanta 600 FEG instrument. Images were acquired through an ETD detector at 30 kV with a spot size 3.5. Electrochemical measurements were performed using a Bio‐Logic 16‐channel VMP‐3 multi‐channel potentiostat/electrochemical impedance spectrometer or a Landt CT3001A battery testing system at room temperature. Electrochemical characterization was carried out using CR2032‐type coin cells. All electrochemical impedance spectroscopy analyses were performed at a range of temperatures from 25 to 60 °C on a Bio‐Logic 16‐channel VMP‐3 multi‐channel potentiostat. Rheological measurements were performed on TA instruments (TA Instruments; New Castle, DE) DHR‐2 stress‐controlled rheometer. A 20 mm cone and plate configuration was used. To measure the viscoelasticity (G’, G’’) of the samples, the frequency sweeps (0.1 to 300 rad s^−1^) were conducted under the constant value of strain amplitude 10% and 25°C. The 10% strain was confirmed to be in the linear viscoelastic region.

### General Procedure for the Synthesis of M2

Exo‐3,6‐epoxy‐1,2,3,6‐tetrahydrophthalic anhydride (0.30 g, 1.8 mmol), poly(ethylene glycol) methyl ether (mPEO‐OH) (i.e., Mn = 9350 g mol^−1^) (1.3 g, 3.7 mmol), and 4‐(dimethylamino)pyridine (22 mg, 0.18 mmol) were added to a flask. The flask was backfilled with nitrogen, and a syringe added anhydrous dichloromethane (60 mL). The resulting solution was stirred and cooled in an ice‐water bath, then *N*‐(3‐dimethylaminopropyl)‐*N*′‐ethylcarbodiimide hydrochloride (EDC·HCl, 0.38 g, 2.0 mmol) in 10 mL of dichloromethane was added to the reaction flask drop‐wise. The water bath was allowed to warm to room temperature overnight. The mixture was washed with water five times. The organic fractions were dried over magnesium sulfate, and the solvent was removed in vacuo to afford an off‐white waxy solid. Note that when higher M_n_ of mPEO‐OH was used to prepare M2 (i.e., M_n_ = 750 or 2000 g mol^−1^), the mixture was purified via two cycles of dialysis (i.e., MWCO 1 KDa for mPEO‐OH, M_n_ = 750 g mol^−1^) in water to afford M2 macromonomers. Water was then removed on a lyophilizer to give a white waxy solid. Yield: 51–58%, ^1^H‐NMR (500 MHz, CDCl_3_): δ 6.46–6.45 (m, 2H), 5.27–5.26 (m, 2H), 4.37–4.16 (m, 4H), 3.81–3.53 (m, H from PEO Unit), 2.84 (s, 6H), 2.84–2.83 (m, 2H). ^13^C NMR (125 MHz, CDCl_3_): δ 171.41, 136.63, 80.61, 71.88, 70.51, 68.91, 64.15, 58.97, 46.81.

### Synthesis of Oxanorbornene Precursor

The synthesis of oxanorbornene precursor was based on a previously reported procedure with minor modifications.^[^ ^89^ ^]^ Maleimide (5.0 g, 52 mmol) and furan (6.0 mL, 82 mmol) were dissolved in toluene (40 mL) at room temperature and then stirred overnight at 80 °C. The mixture was then cooled to ambient temperature, and the flask was put in the ice bath, forming white precipitates. The solids were isolated by vacuum filtration, washed with diethyl ether, and dried under vacuum to yield the final product as white powders. Yield: 93%, ^1^H‐NMR (500 MHz, DMSO‐d_6_): δ 11.14 (s, 1H), 6.53 (t, *J* = 1.0 Hz, 2H), 5.11 (t, *J* = 0.9 Hz, 2H), 2.84 (s, 2H). ^13^C NMR (125 MHz, DMSO‐d_6_): δ 178.28, 136.92, 80.76, 48.87.

### Synthesis of M1

The synthesis of M1 was based on a previously reported procedure with minor modifications.^[^ ^90^ ^]^ The oxanorbornene precursor (2.0 g, 12 mmol), mPEO‐OH, M_n_ = 1000 g mol^−1^ (7.1 g, 7.1 mmol), and triphenylphosphine (2.2 g, 8.6 mmol) were added to a flask. The flask was backfilled with nitrogen, and anhydrous tetrahydrofuran (60 mL) was added by a syringe. The resulting solution was stirred and cooled in an ice–water bath. Diiospropyl azodicarboxylate (DIAD, 1.4 mL, 8.6 mmol) was added dropwise to the above solution using an additional funnel. The solution was brought to room temperature slowly and stirred overnight. The solvent was evaporated under reduced pressure, and the residue was dissolved in water. The aqueous solution was washed with ethyl acetate to remove unreacted monomer, mPEO‐OH, reduced DIAD, and triphenylphosphine oxide. Water was then removed on a lyophilizer to give a white solid. Yield: 84%, ^1^H‐NMR (500 MHz, CDCl_3_): δ 6.51 (s, 2H), 5.26 (s, 2H), 3.79‐3.48 (m, H from PEO Unit), 3.38 (s, 3H), 2,86 (s, 2H). ^13^C NMR (125 MHz, CDCl_3_): δ 176.05, 136.52, 80.85, 71.91, 70.54, 67.08, 58.99, 47.44, 38.16.

### Synthesis of P2 and P1

Under an inert gas atmosphere, Grubbs third generation catalyst (G3) was dissolved in a minimal amount of freeze‐pump‐thawed dichloromethane and added quickly to a solution of M2 or M1 macromonomer in dichloromethane ([monomer] = 0.14 −0.04 m, from [monomer]:[cat] = 1:10 to [monomer]:[cat] = 1:300). The resulting solution was stirred for 2 or 18 h depending on the completion of polymerization. Excess ethyl vinyl ether was added to the flask, stirring the solution for 2 h. The solvent was removed under a vacuum to afford a faint brown solid. Purification was performed by repeated precipitation in hexane/ethyl ether = 1:1 (v/v), afforded a faint yellow solid or off‐white solid. Yield: 90%–93%. ^1^H‐NMR of P2 (400 MHz, CDCl_3_): ∂ 5.94–5.81 (br, m), 5.61–5.49 (br, m), 5.18–4.99 (br, m), 4.74–4.59 (br, m), 4.33–4.11 (br, m), 3.80–3.48 (br, m), 3.41–3.35 (br, m), 3.10–3.01 (br, m). ^1^H‐NMR of P1 (400 MHz, CDCl_3_): ∂ 6.12–6.02 (br, m), 5.85–5.70 (br, m), 5.13–4.90 (br, m), 4.50–4.35 (br, m), 3.80–3.45 (br, m), 3.41–3.35 (br, m), 3.35–3.24 (br, m).

### Synthesis of N‐M1

The synthesis of norbornene precursor was based on a previously reported procedure with minor modifications.^[^ ^91^ ^]^ A round flask with a condenser was charged with *cis*‐norbornene‐*exo*‐2,3‐dicarboxylic anhydride (3.0 g, 18 mmol) and urea (1.2 g, 20 mmol). The mixture in the flask was placed in an oil bath for stirring (160 °C) for 1 h and then cooled to room temperature. Boiling water (10 mL) was added to the flask after cooling down the reaction flask to 80 °C, and the suspension was stirred at the same temperature until all the solids were dissolved (it would take ≈ 30 min). Then, recrystallize the compound in the same flask by leaving it overnight upon cooling. The norbornene precursor was obtained by vacuum filtration as faint yellow crystals. Yield: 64%. ^1^H‐NMR (400 MHz, CDCl_3_): ∂ 8.37 (s, 1H), 6.27 (t, *J* = 1.9 Hz, 2H), 3.28 (p, *J* = 1.7 Hz, 2H), 2.72 (d, *J* = 1.4 Hz, 2H), 1.59–1.52 (m, 1H), 1.49–1.42 (m, 1H). ^13^C NMR (125 MHz, CDCl_3_): δ 178.44, 137.74, 77.06, 49.16, 45.12, 42.89.

Then, the norbornene precursor (0.22 g, 1.3 mmol), mPEO‐OH, M_n_ = 1000 g mol^−1^ (1.2 g, 1.2 mmol), and triphenylphosphine (0.38 g, 1.4 mmol) were added to a flask. The flask was backfilled with nitrogen, and anhydrous tetrahydrofuran (30 mL) was added by a syringe. The resulting solution was stirred and cooled in an ice–water bath. Diisopropyl azodicarboxylate (DIAD, 0.24 mL, 1.4 mmol) was added dropwise to the above solution using an additional funnel. The solution was brought to room temperature and stirred overnight. The solvent was evaporated under reduced pressure, and the residue was dissolved in water. The aqueous solution was washed with ethyl acetate to remove unreacted monomer, mPEO‐OH, reduced DIAD, and triphenylphosphine oxide. Water was then removed on a lyophilizer to give a white solid. Yield: 79%, ^1^H‐NMR (500 MHz, CDCl_3_): δ 6.27 (m, 2H), 3.78–3.52 (m, H from PEO Unit), 3.37 (s, 3H), 3.27–3.24 (m, 2H), 1.49–1.45 (m, 1H), 1.38–1.34 (m, 1H). ^13^C NMR (125 MHz, CDCl_3_): δ 177.92, 137.80, 71.92, 70.59, 69.86, 66.86, 59.00, 47.79, 45.25, 42.69, 37.72.

### Synthesis of Nor‐P1

Under an inert gas atmosphere, Grubbs third generation catalyst (G3) was dissolved in a minimal amount of freeze‐pump‐thawed dichloromethane and added quickly to a solution of Nor‐M1 macromonomer in dichloromethane ([monomer] = 0.04 m, [monomer]:[cat] = 1:150). The resulting solution was stirred for 3 h, and excess ethyl vinyl ether was added to the flask and stirred for 2 h. The solvent was removed under vacuum to afford a faint brown solid. Purification was performed by repeated precipitation in hexane/ethyl ether = 1:1 (v/v), afforded an off‐white solid. Yield: 93%. ^1^H‐NMR (400 MHz, CDCl_3_): ∂ 5.83–5.68 (br, m), 5.63–5.49 (br, m), 3.81–3.46 (br, m), 3.41–3.35 (br, m), 3.33–3.25 (br, m), 3.16–2.88 (br, m), 2.86–2.60 (br, m), 2.36–2.00 (br, m), 1.69–1.48 (br, m).

### Preparation of P1 and P2 Polymer Electrolytes

LiTFSI and P1 or P2 polymer were mixed in a certain ratio in anhydrous tetrahydrofuran and stirred for 1 h. Typically, the average molar ratio of ethylene oxide units (EO) to lithium‐ions was kept to 10/1. Then the mixed solution was drop‐cast onto a substrate (stainless steel or lithium metal) in the glovebox (level of H_2_O and O_2_ < 1 ppm). A Teflon ring with a thickness of 0.8 mm was used so that the thickness of the electrolyte remains relatively constant between the samples. The THF was evaporated on a hot plate with a temperature of 40 °C for 1 h. Afterward, the samples were placed into the glovebox's vacuum chamber and dried under vacuum for 3 h. Note that the total amount of THF used per sample (or battery) is < 0.5 mL.

### Ionic Conductivity Measurements

The ionic conductivity measurements of the polymer electrolytes were carried out using the AC impedance spectroscopic technique. The polymer electrolytes were sandwiched as Stainless steel/P1 or P2 electrolytes/Stainless steel in a glovebox with an argon atmosphere (level of H_2_O and O_2_ < 1 ppm). The measurements were performed at temperatures ranging from 25 to 60 °C. The cell was thermally equilibrated for 30 min at each temperature point before measurements. The AC impedance spectra were recorded over the 0.01–10^6^ Hz frequency range with a voltage amplitude of 100 mV. The ionic conductivity values were derived from the measured resistance using Equation S1 (Supporting Information).

### Linear Sweep Voltammetry (LSV) curves

The electrochemical window stability of the polymer electrolyte was evaluated using Li/SPE/stainless steel (asymmetric battery configuration) by sweeping the voltage from 3.0 to 5.5 V with a scan rate of 0.1 mV s^−1^.

### Electrode Preparation and Coin Cell Assembly

Electrochemical characterization was carried out using 2032‐type coin cell. LFP active material was combined with carbon Super P and PVDF in an 80:10:10 (w/w/w) ratio and suspended in NMP at 0.05 g mL^−1^ concentration. The slurry was sonicated for 30 min, stirring every 10 min to homogenize. The slurry was then loaded into a spray gun (Master, Airbrush, G22). With a constant airflow, the liquid was sprayed uniformly on an aluminum foil preheated to 150 °C. The as‐obtained coated cathode film was taken off from the heating plate and dried at 80 °C in a vacuum oven overnight. An electrode punch was used to cut the electrodes to a 10 mm diameter (0.9 mg cm^−2^ active material loading on the current collector). Lithium foil with a diameter of 11 mm was used as the anode. The coin cells were assembled using P1 or P2 polymer electrolyte, a stainless spacer (0.5 mm thickness), and one spring for optimal cell compression. The cells were hermetically sealed using a pressure‐controlled electric crimper (MTI Corporation). For Li/SPE/Li cell configuration, SPE P1 or P2 was sandwiched between two 11 mm diameters of lithium electrodes. The electrolyte was allowed to have good contact with the electrodes for 10 h before cycling all batteries. Prior to the cycling experiment, the battery was subjected to three formation cycles at 0.1 C‐rate from 2.5 to 3.8 V. Rate test and long‐term cycling testing of Li|SPE|LFP cell were conducted on BioLogic channels at room temperature. For post‐mortem analysis of cycled cells, coin cells were disassembled in a glovebox, then the lithium electrode with polymer electrolyte was cut into halves using a blade. The electrodes with the polymer electrolyte layer were directly used for SEM analysis.

## Conflict of Interest

The authors declare no conflict of interest.

## Author Contributions

S.Y.A. conceived the initial idea and created the synthetic design. The research design was conceptualized by S.Y.A., J.F.W., and K.M. S.Y.A. performed the syntheses, material characterization, and electrochemical characterization. X.W. and T.L. assisted with the electrochemical characterization. Y.Z. and R.Y. assisted with the polymer characterization. J.H.A. and L.M.W. ran the rheometer experiments/optimization. All authors contributed to the data analysis and writing the paper.

## Supporting information

Supporting Information

## Data Availability

The data that support the findings of this study are available from the corresponding author upon reasonable request.
