# Supplementary material for: Highly Conductive Polyoxanorbornene‐Based Polymer Electrolyte for Lithium‐Metal Batteries
Source: Adv Sci (Weinh). 2023 Jul 17;10(27):2302932. doi: 10.1002/advs.202302932 (PMC10520635; doi:10.1002/advs.202302932)
Supplement: Supplementary file 1 — Supporting Information [file ADVS-10-2302932-s001.pdf]

ADVANCED SCIENCE
Open Access

Supporting Information

for *Adv. Sci.*, DOI 10.1002/advs.202302932

Highly Conductive Polyoxanorbornene-Based Polymer Electrolyte for Lithium-Metal Batteries

So Young An, Xinsheng Wu, Yuqi Zhao, Tong Liu, Rongguan Yin, Jung Hyun Ahn, Lynn M. Walker, Jay F. Whitacre and Krzysztof Matyjaszewski**

Supporting Information

Highly Conductive Polyoxanorbornene-Based Polymer Electrolyte for Lithium-Metal Batteries

So Young An[a], Xinsheng Wu[b], Yuqi Zhao[b], Tong Liu[a], Rongguan Yin[a], Jung Hyun Ahn[d], Lynn M. Walker[d], Jay. F. Whitacre[b,c]* and Krzysztof Matyjaszewski[a]*

[a] Department of Chemistry, Carnegie Mellon University, 4400 Fifth Avenue, Pittsburgh, Pennsylvania, 15213, United States
[b] Department of Materials Science and Engineering, Carnegie Mellon University, 5000 Forbes Avenue, Pittsburgh, Pennsylvania, 15213, United States
[c] Scott Institute for Energy Innovation, Carnegie Mellon University, 5000 Forbes Avenue, Pittsburgh, Pennsylvania, 15213, United States
[d] Department of Chemical Engineering, Carnegie Mellon University, 5000 Forbes Avenue, Pittsburgh, Pennsylvania, 15213, United States

Table S1. Summary of M1 and M2 syntheses.

**Entry**	**Starting Oxanorbornene**	**OH-mPEO$_{ave}$ M$_n$ (g/mol)**	**Resulting M2$_{ave}$ (g/mol)**
M2-1	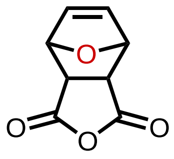	350	912
M2-2		1000	2210
M2-3		2000	4060
M1	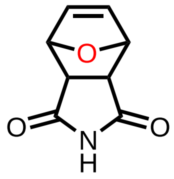	1000	1147

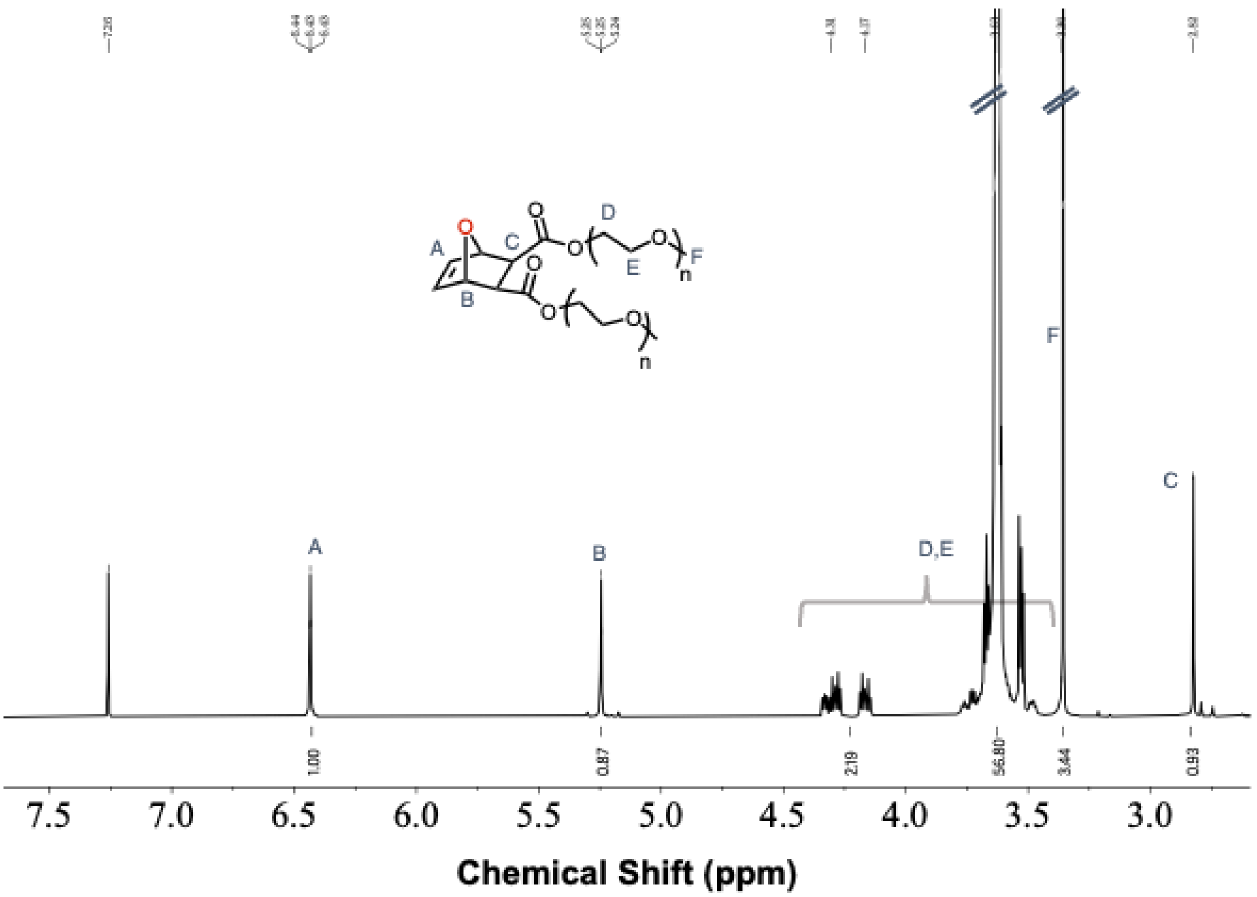

Figure S1. Representative ^{1}H-NMR spectrum of M2 ($M_{n\ ave}$= 912 g/mol).

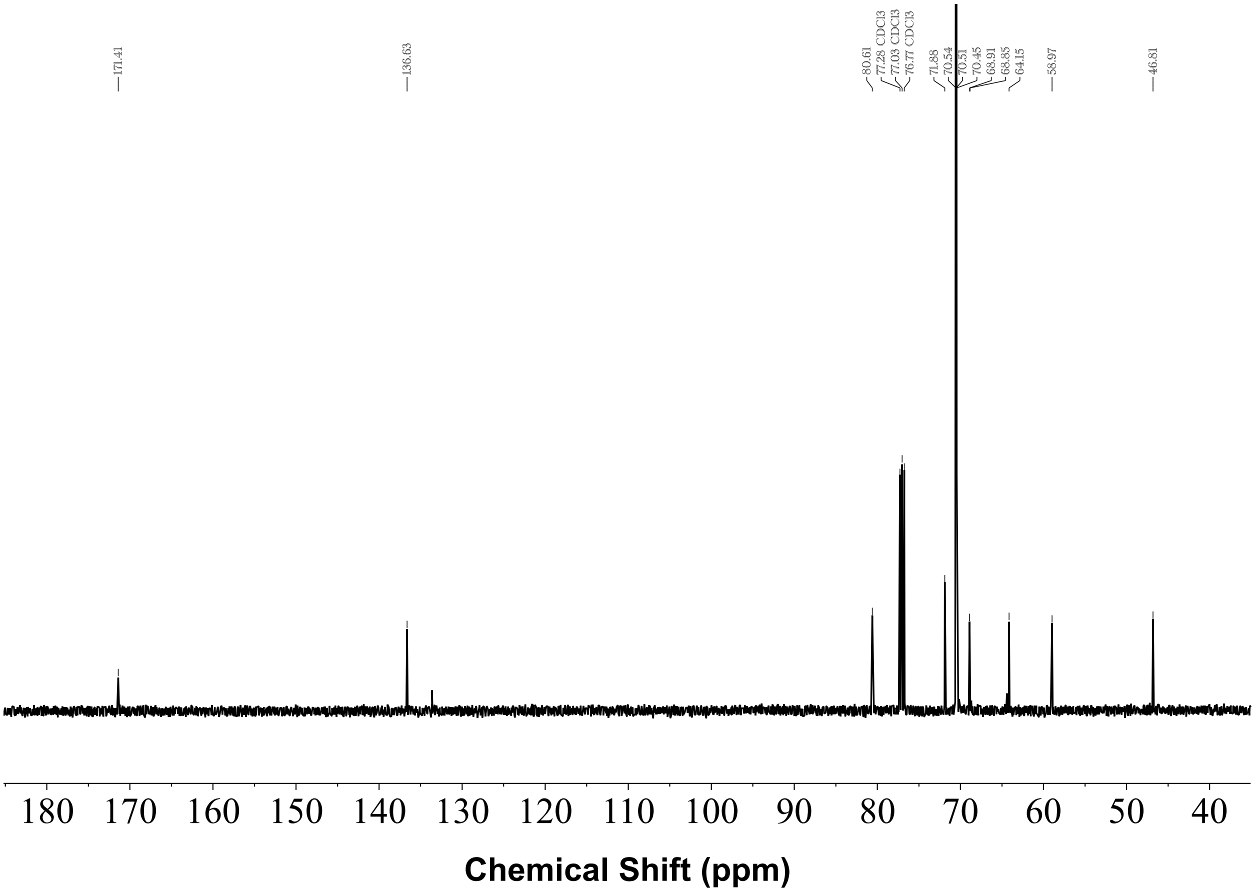

Figure S2. Representative ^{13}C-NMR spectrum of M2 ($M_{n\ ave}$ = 912 g/mol).

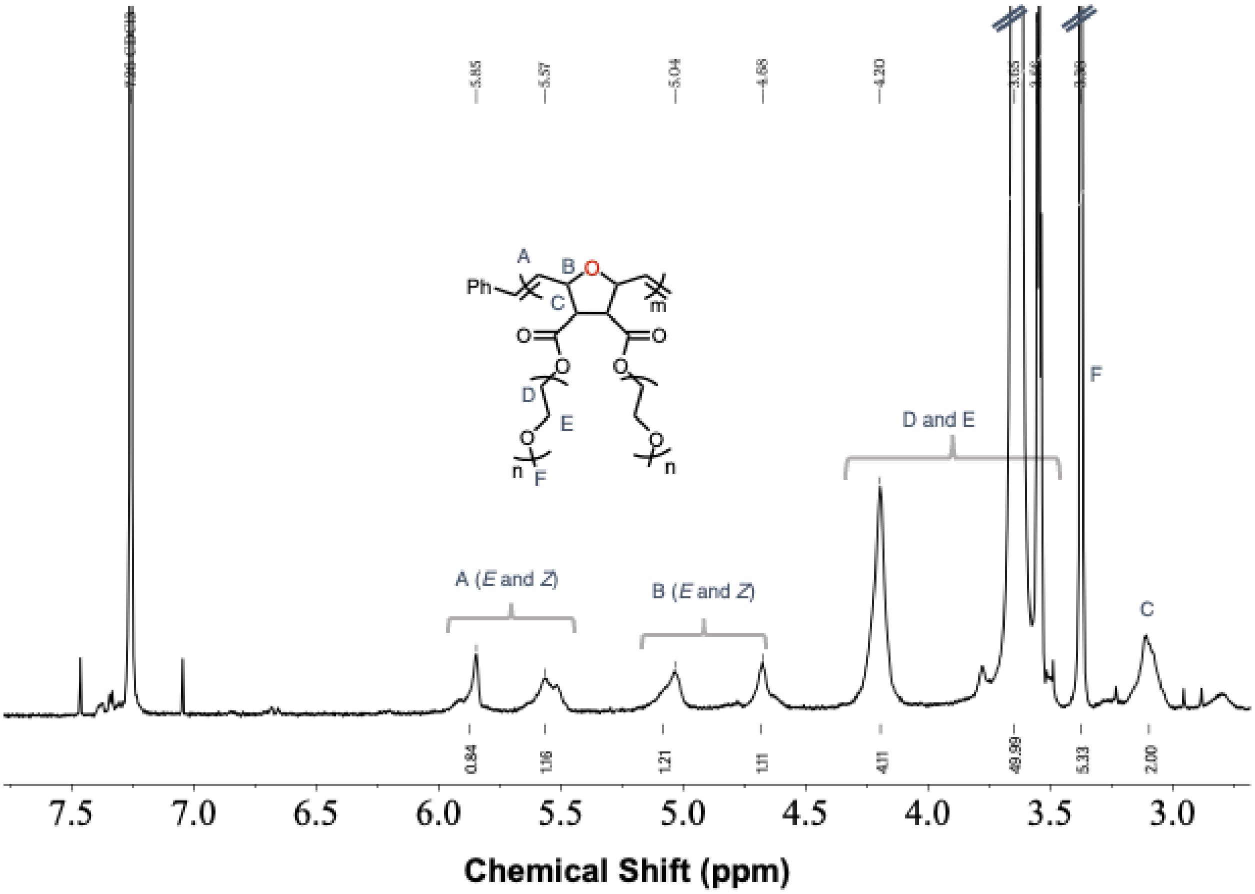

Figure S3. Representative ^{1}H-NMR spectrum of P2.

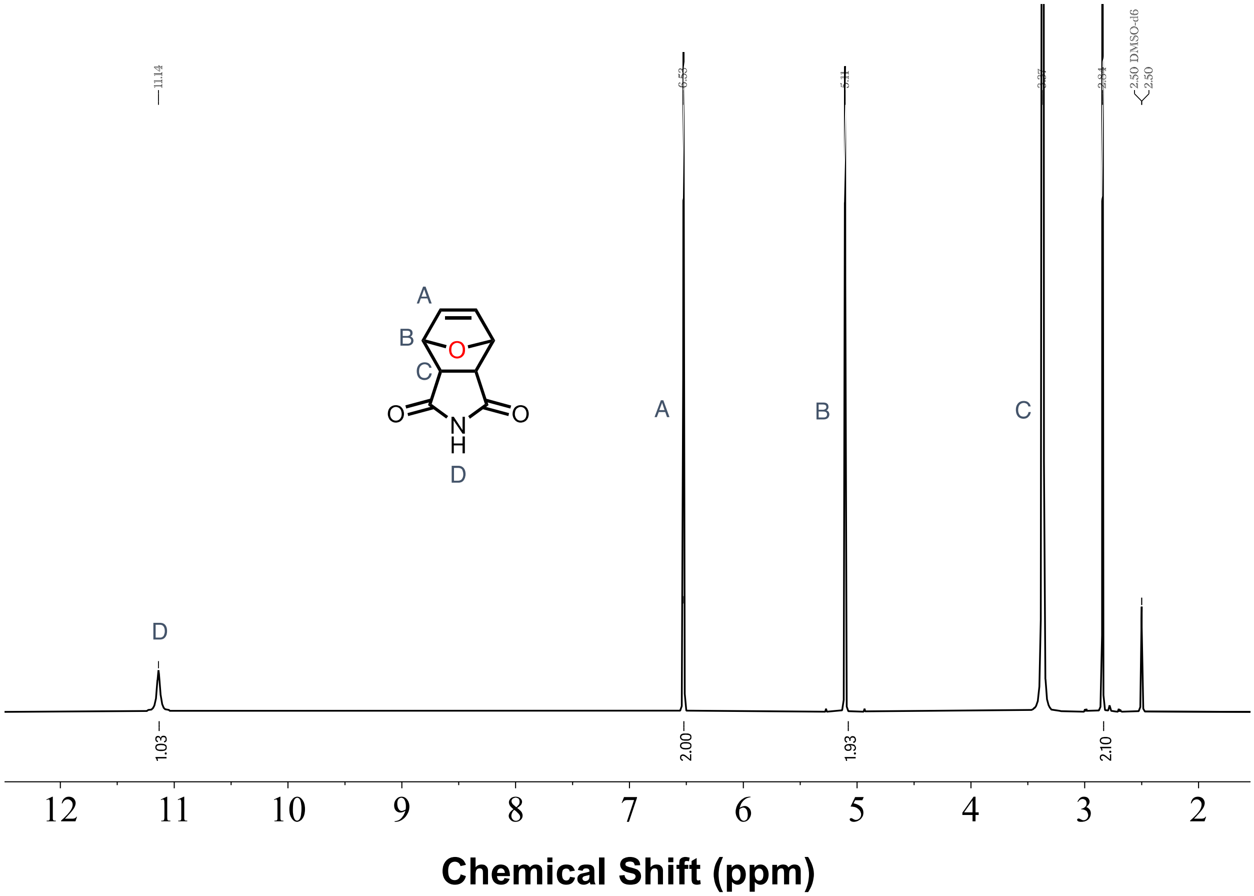

Figure S4. ^{1}H-NMR spectrum of oxanorbornene precursor.

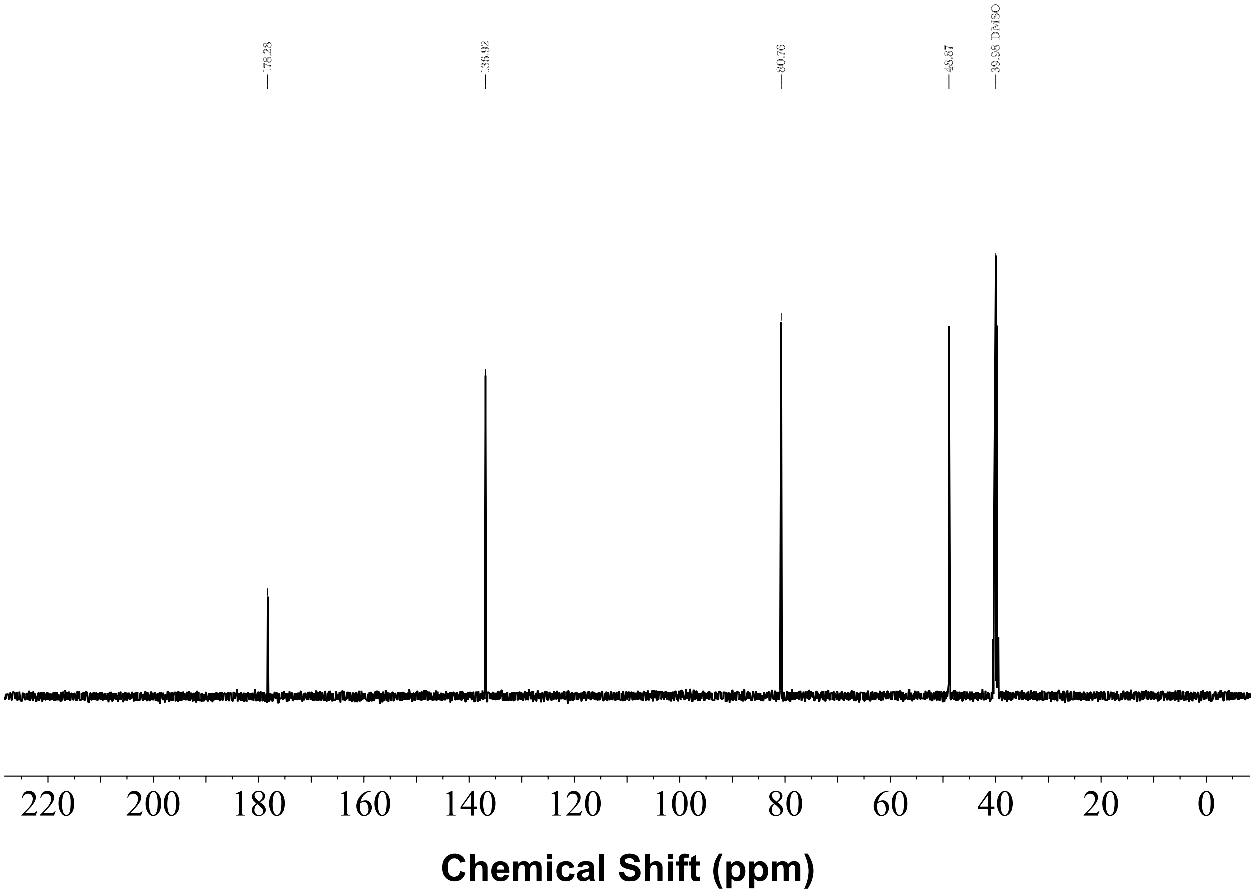

Figure S5. ^{13}C-NMR spectrum of oxanorbornene precursor.

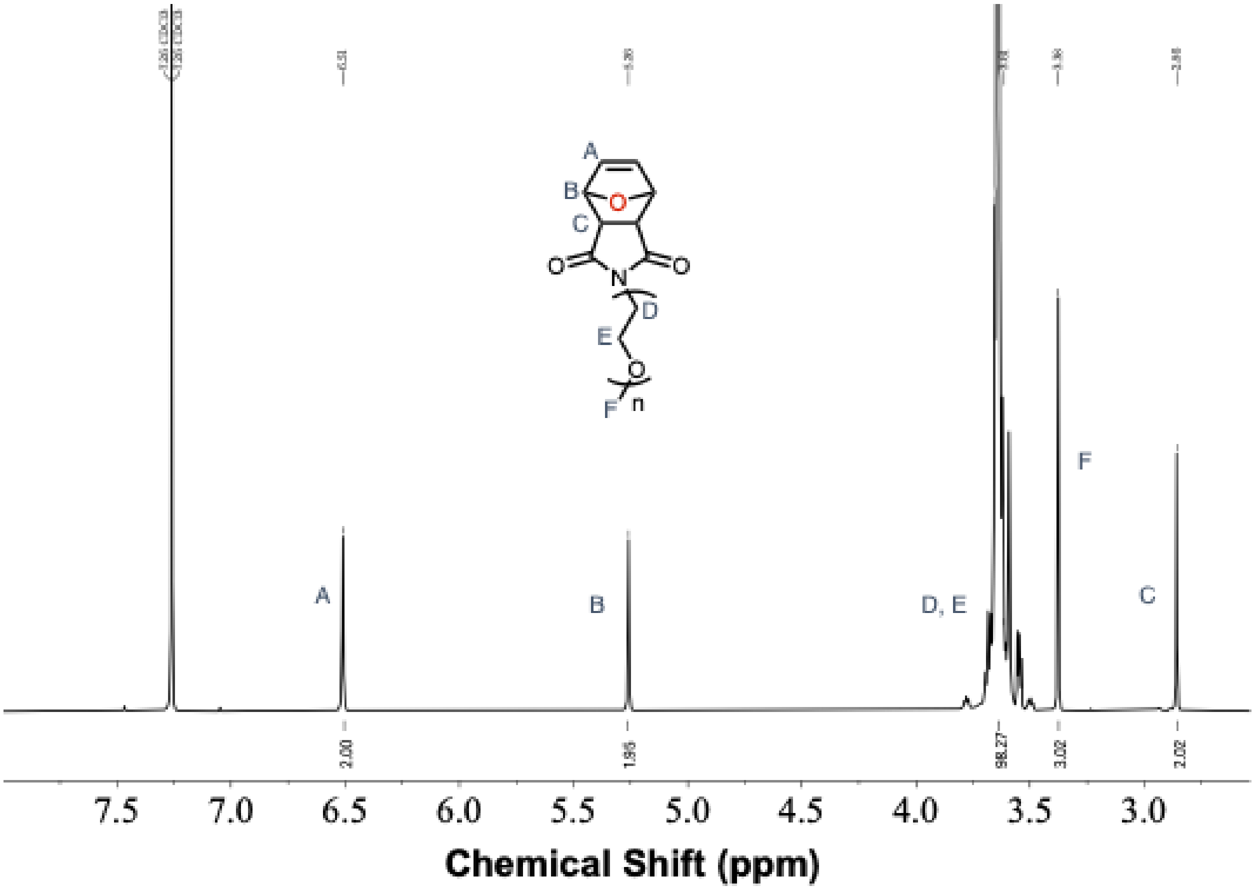

Figure S6. ^{1}H-NMR spectrum of M1.

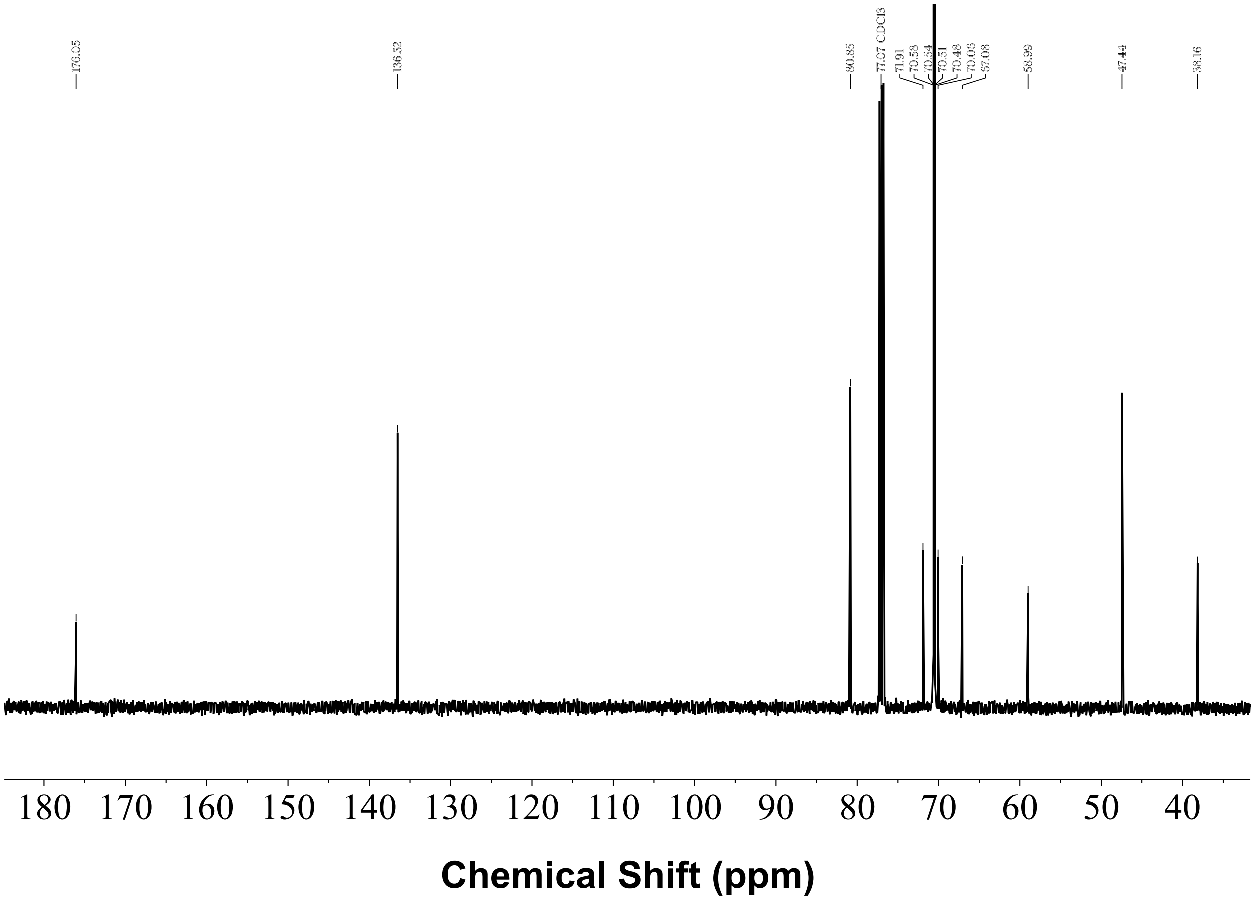

Figure S7. ^{13}C-NMR spectrum of M1.

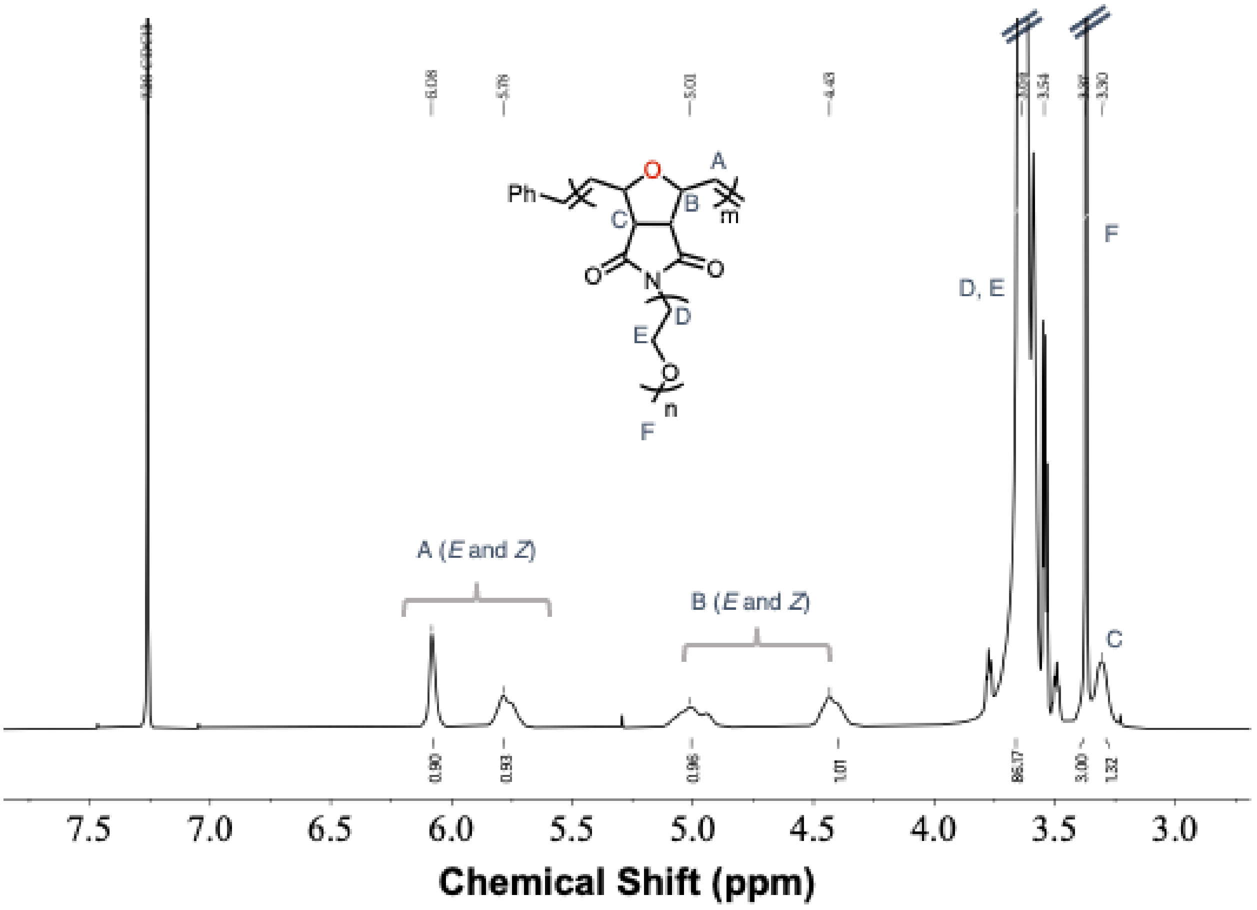

Figure S8. Representative ^{1}H-NMR spectrum of P1.

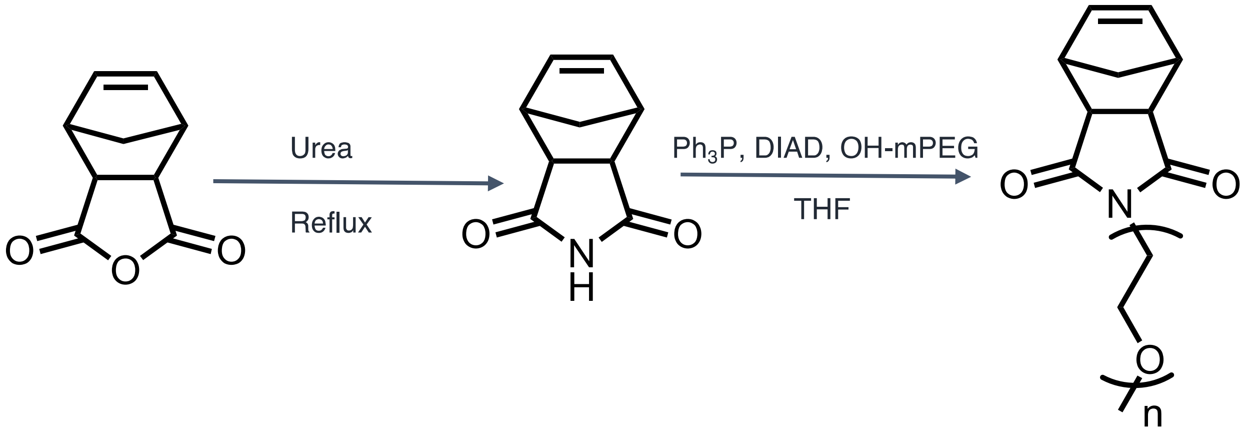

Figure S9. Synthesis of norbornene-based macromonomer (Nor-M1).

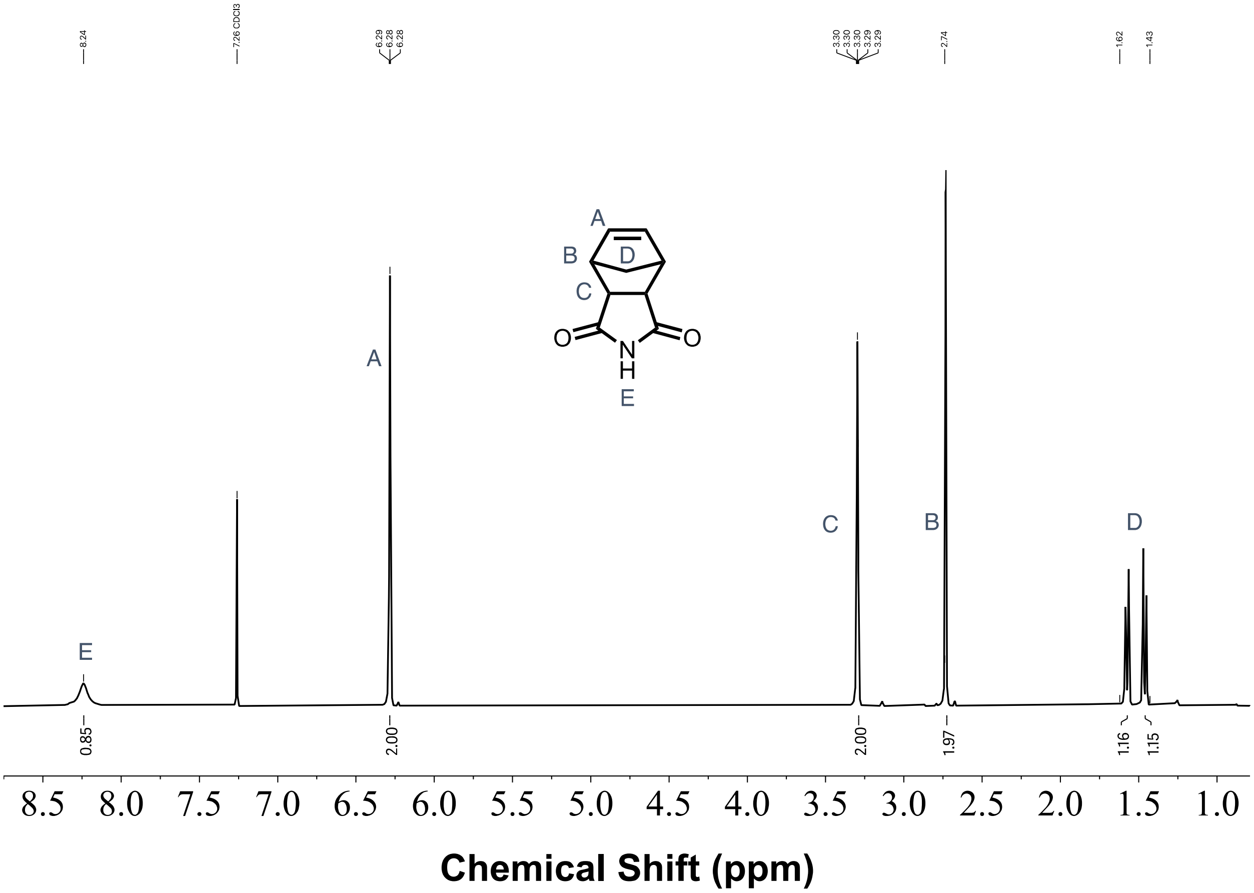

Figure S10. ^{1}H-NMR spectrum of norbornene precursor.

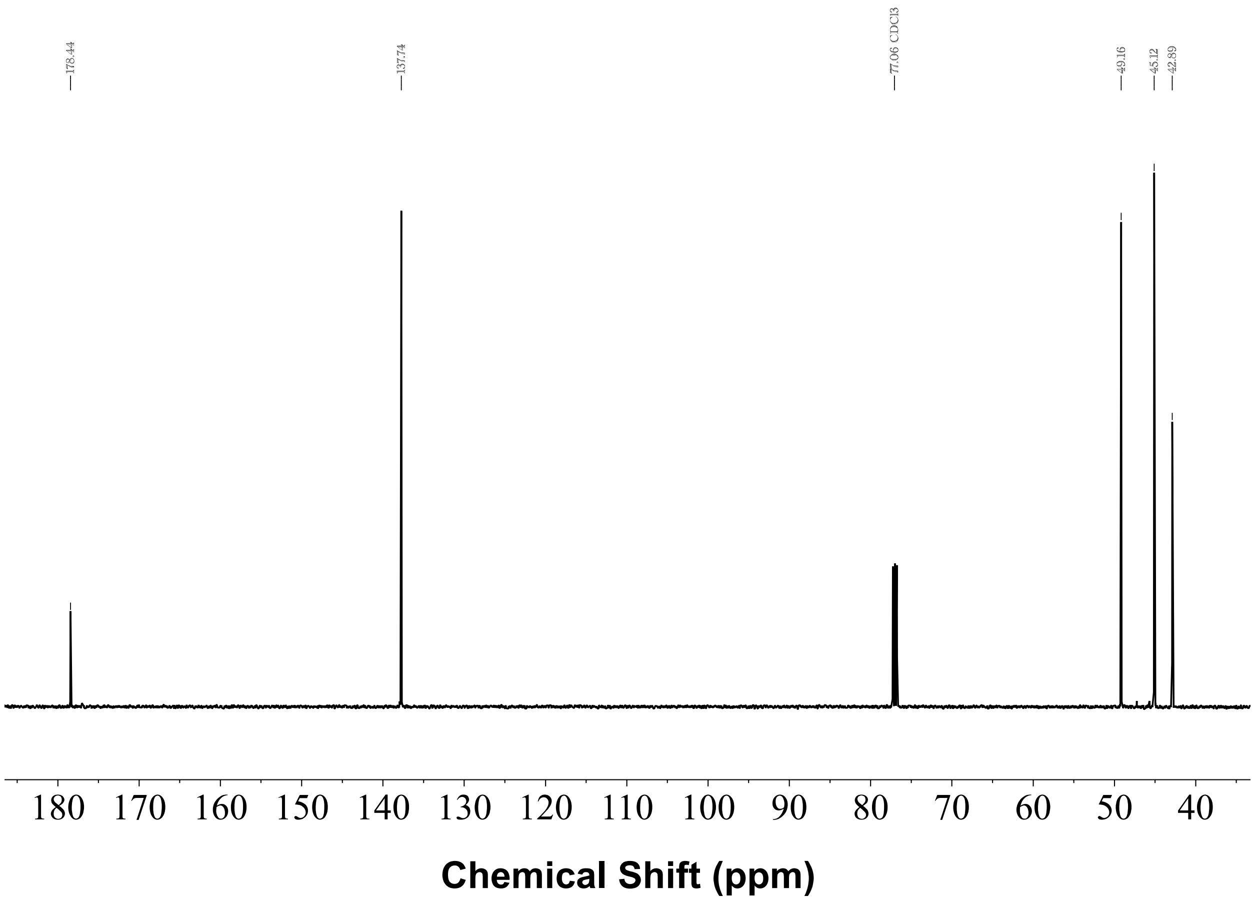

Figure S11. ^{13}C-NMR spectrum of norbornene precursor.

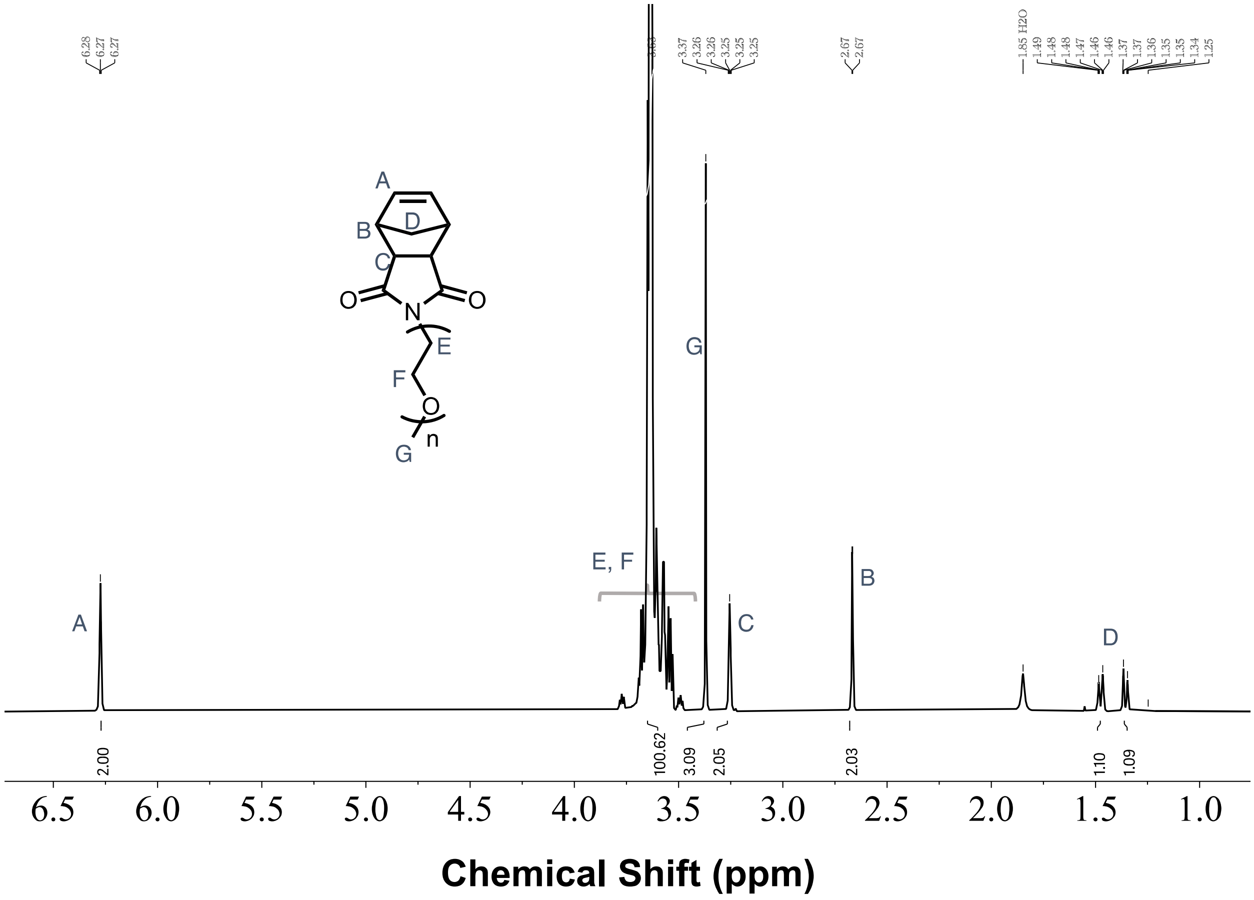

Figure S12. ^{1}H-NMR spectrum of Nor-M1.

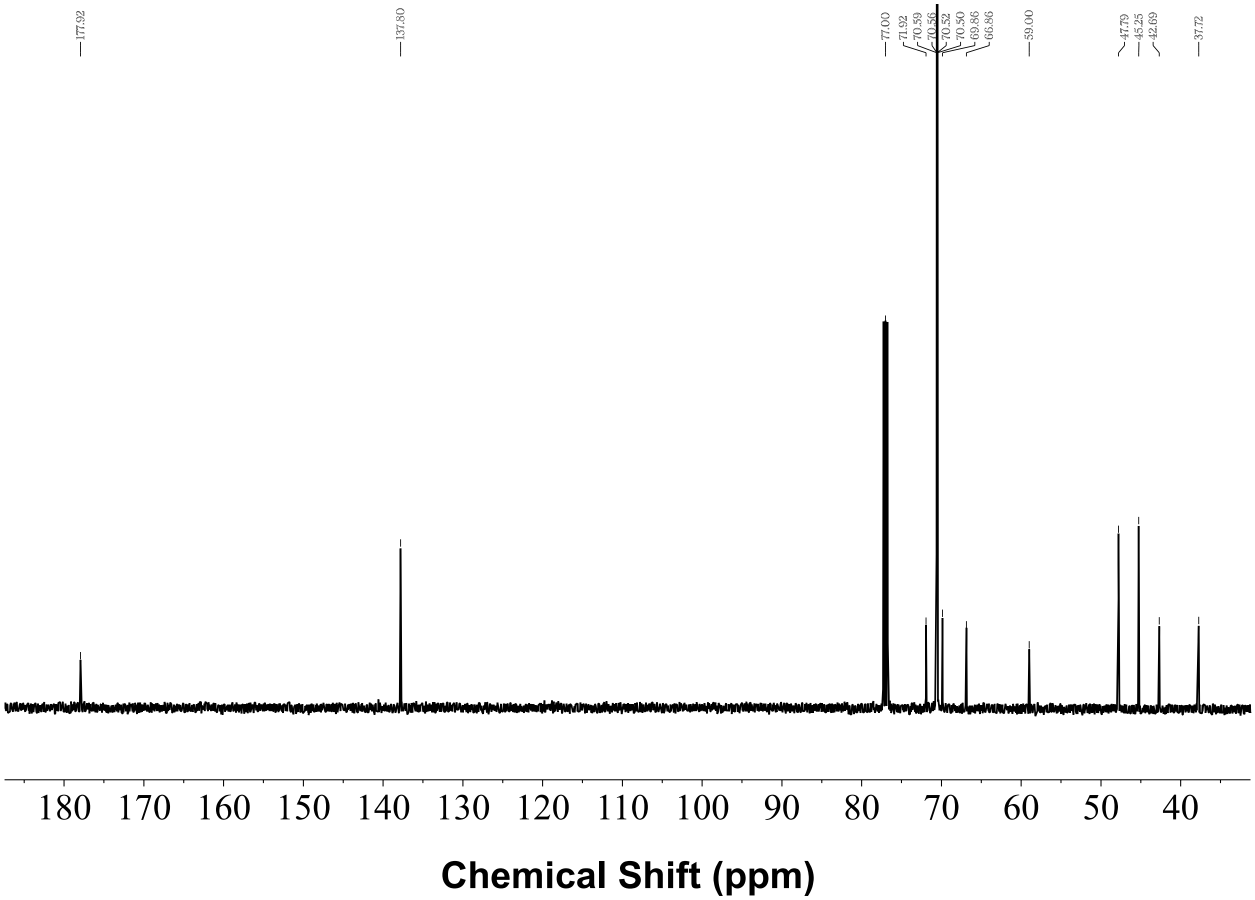

Figure S13. ^{13}C-NMR spectrum of Nor-M1.

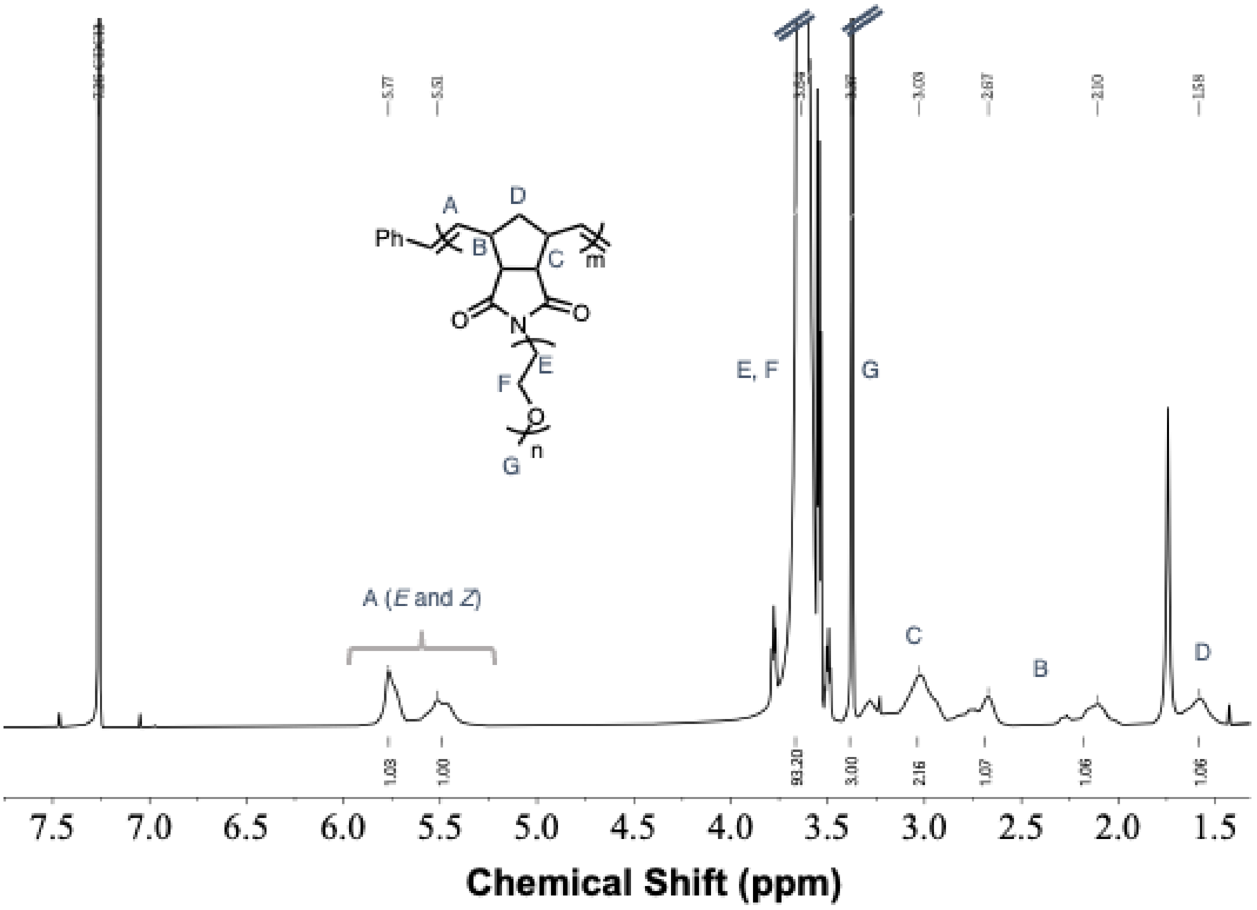

Figure S14. ^{1}H-NMR spectrum of Nor-P1.

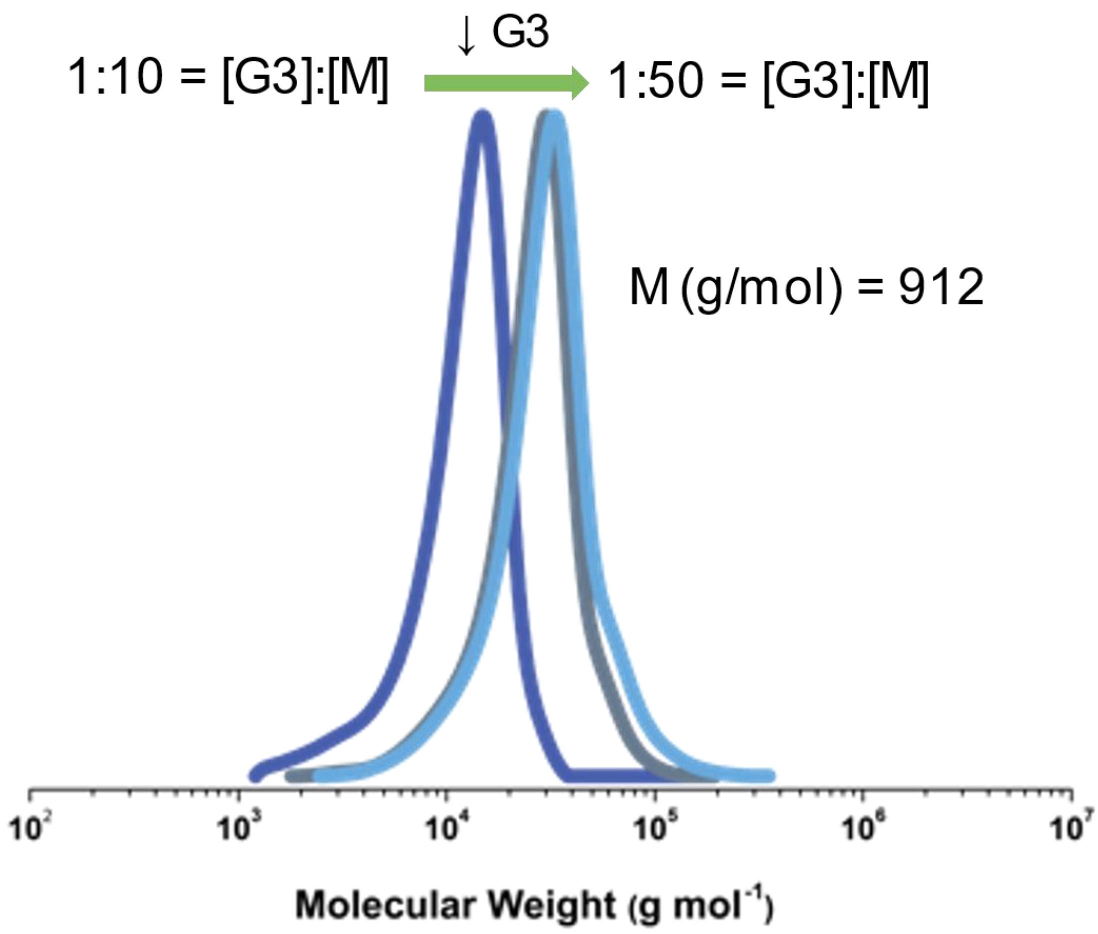

Figure S15. GPC traces of P2 with different $M_{n\ ave}$ = 912 g/mol of M1

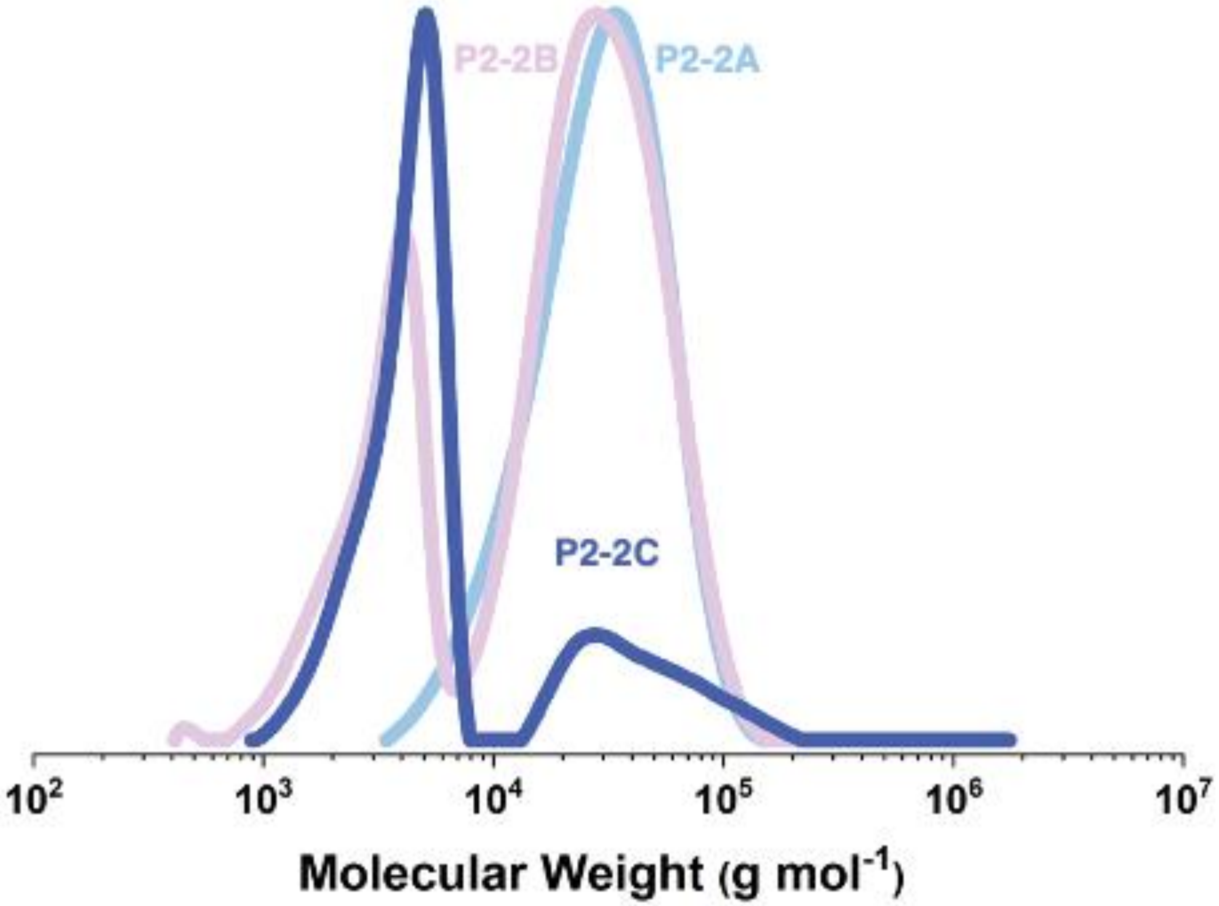

Figure S16. GPC traces of P2 with different $M_{n\ ave}$ = 2210 g/mol of M1

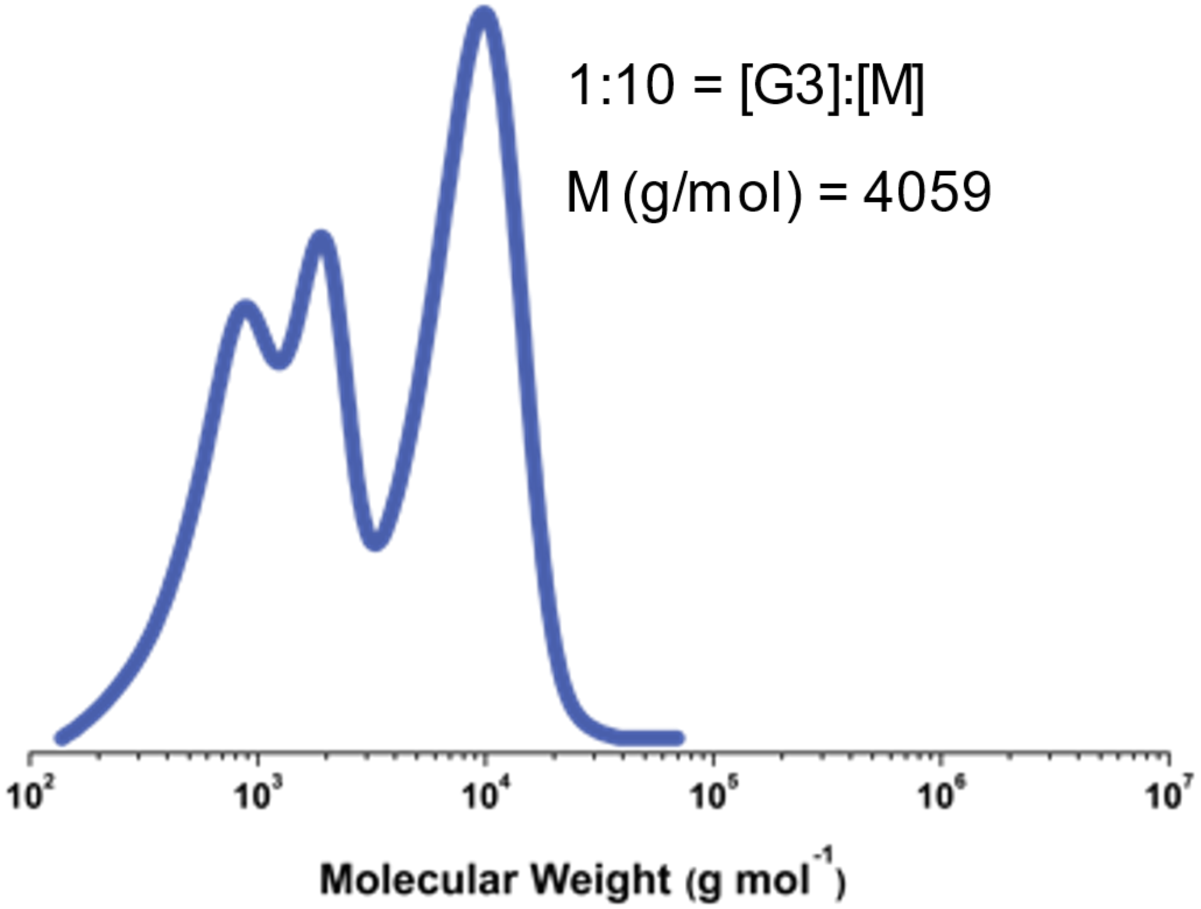

Figure S17. GPC traces of P2 with different $M_{n\ ave}$ = 4060 g/mol of M1.

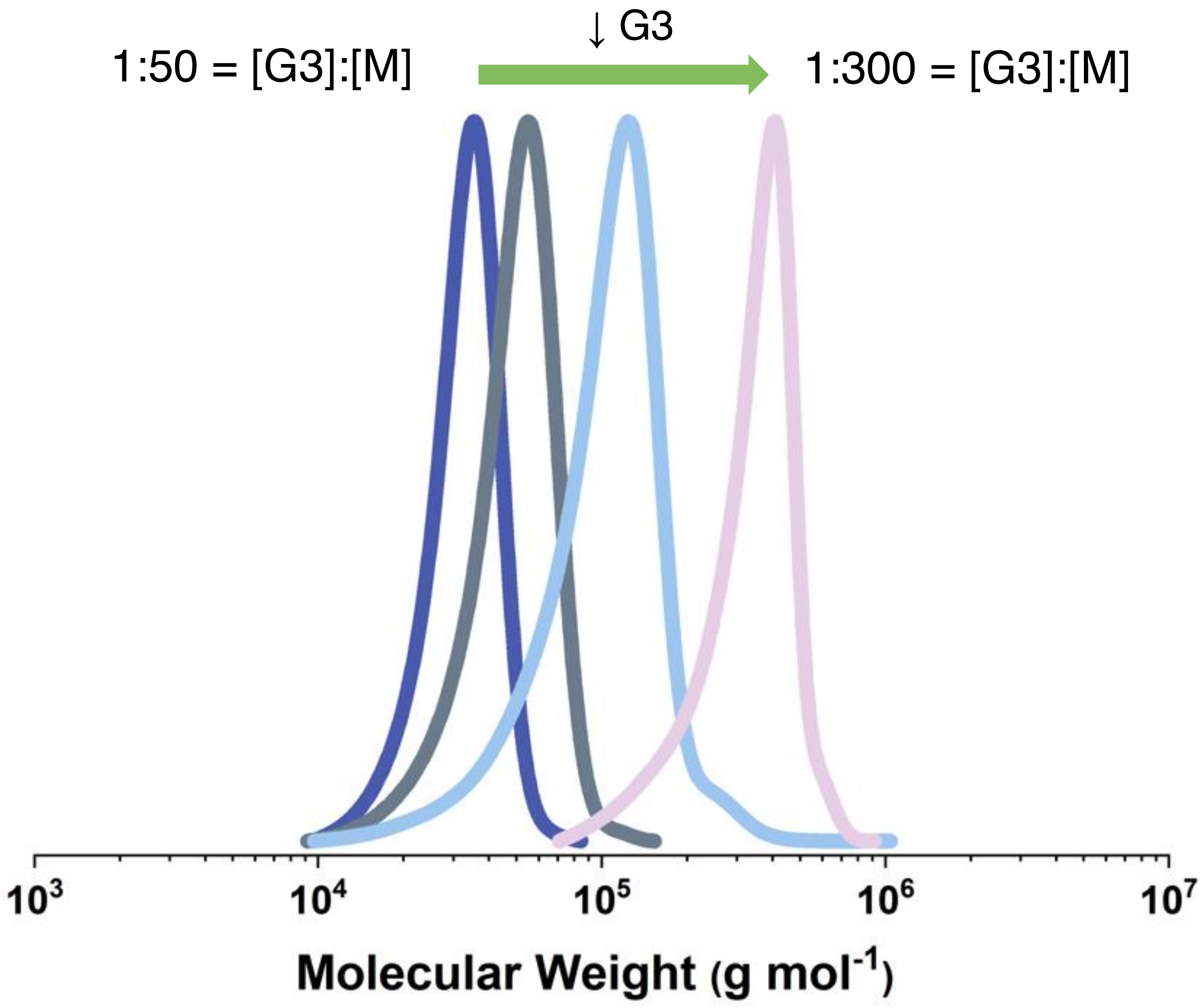

Figure S18. GPC traces of P1.

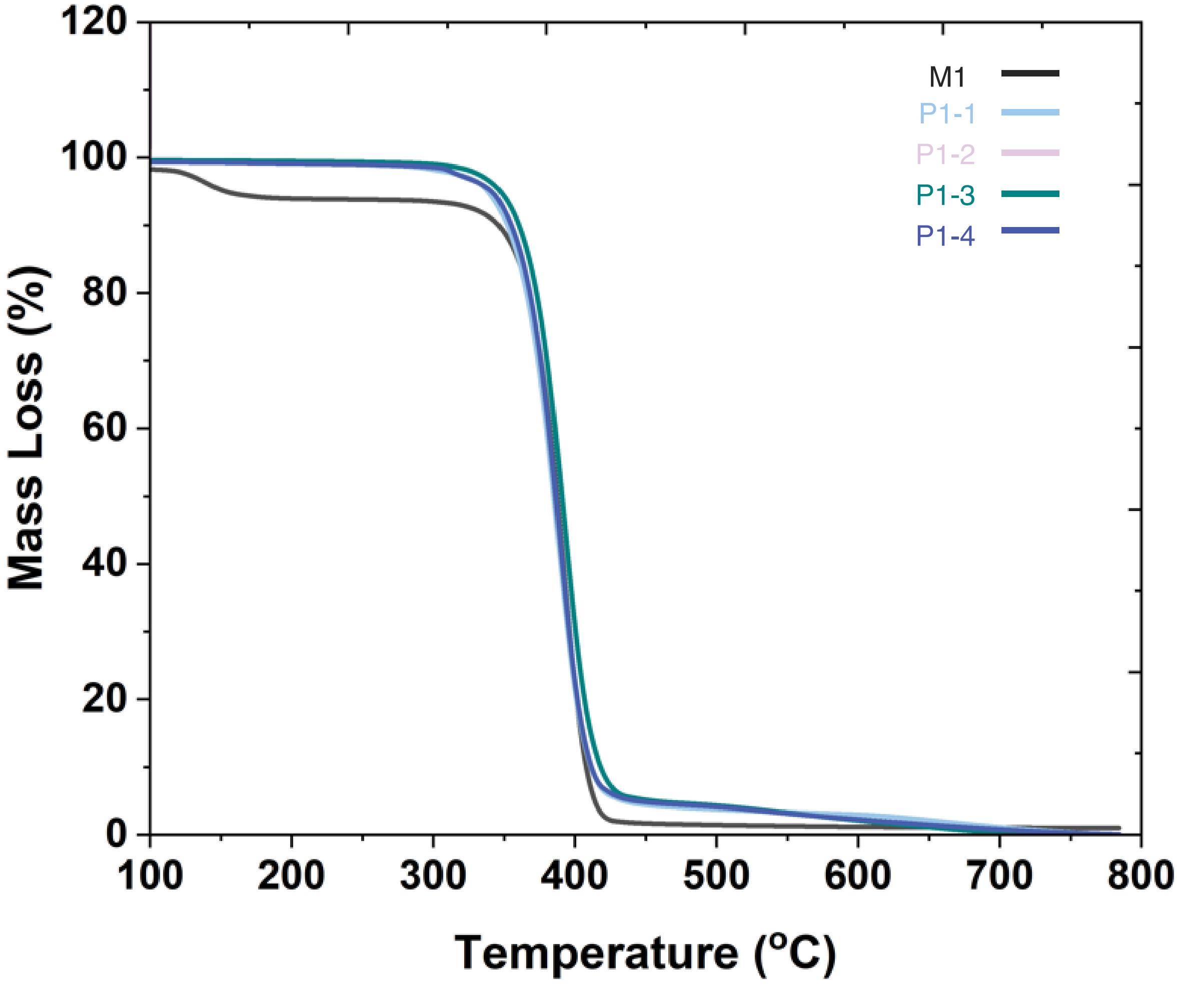

Figure S19. Thermal properties of M1, P1-1, P1-2, P1-3 and P1-4.

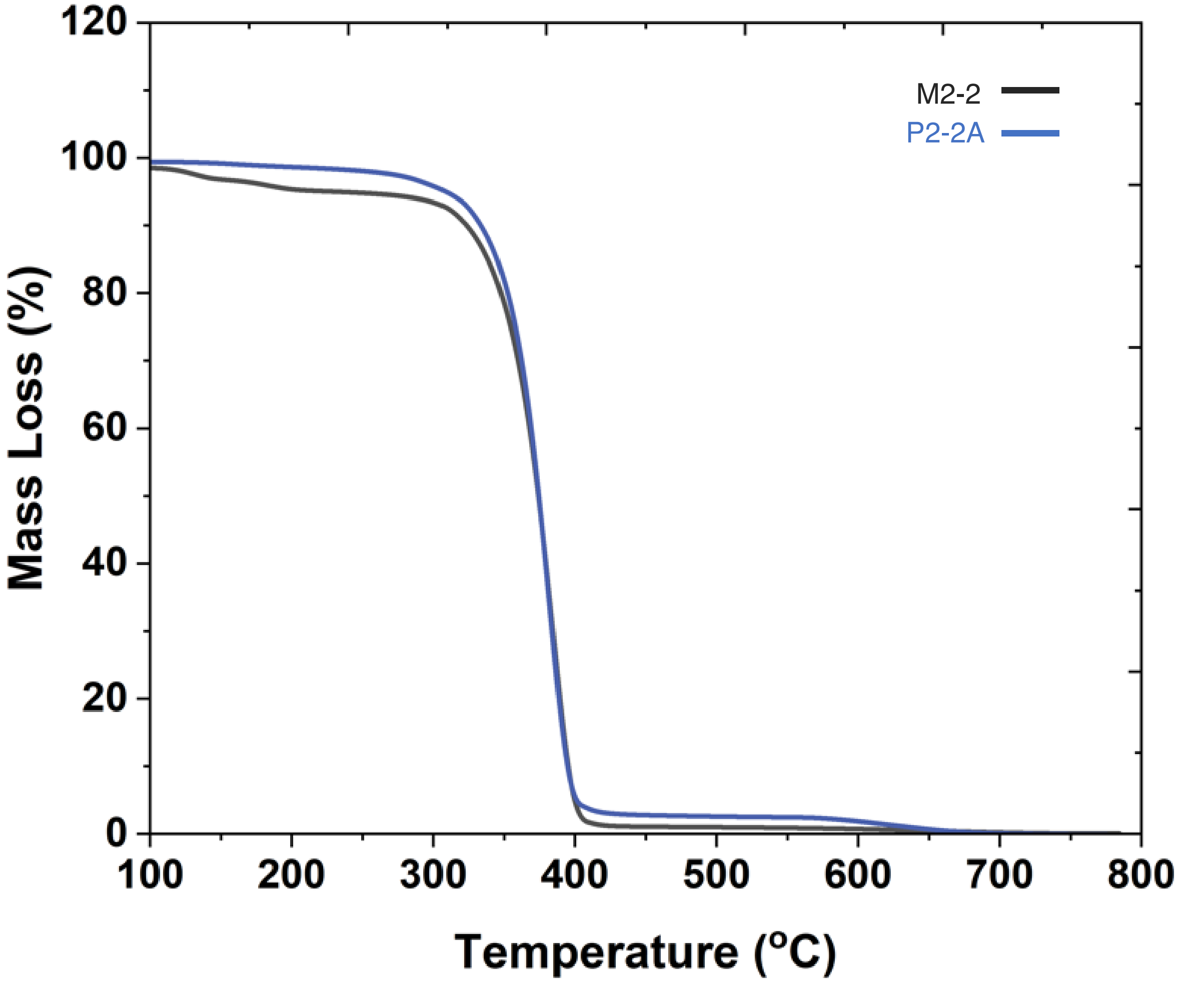

Figure S20. Thermal properties of M2-2 and P2-2A

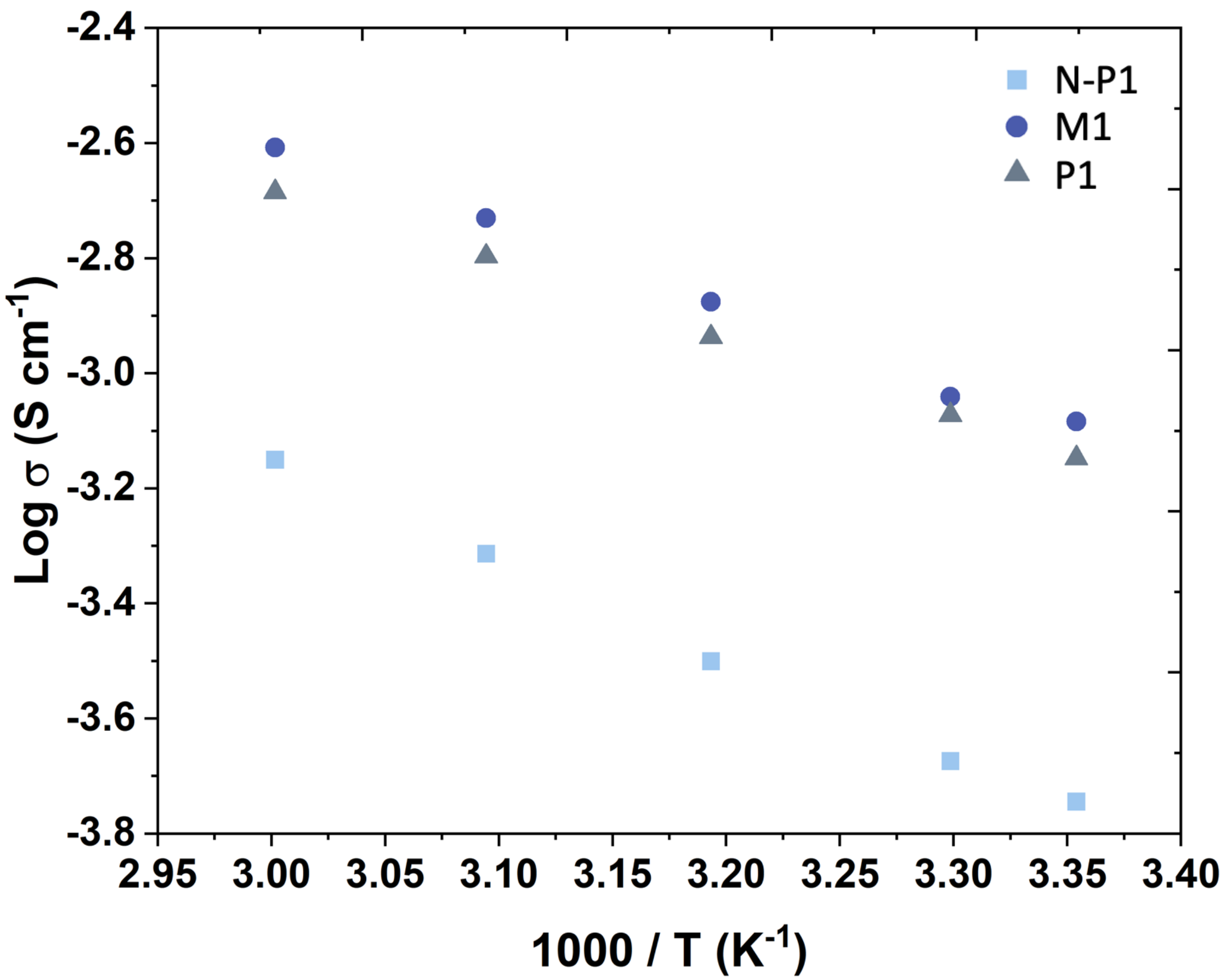

Figure S21. Ionic conductivity of N-P1 and M1, compared to P1 with DP 150.

Table S2. Summary of ionic conductivity for M1 and N-P1.

Entry	M1 or N-M1 (g/mol)	DP_{bb}	DP_{sc}	σ (S cm^{-1}) at RT	σ (S cm^{-1}) at 60 ^{o}C
P1-3	1147	150	23	7.12×10^{-4}	2.05×10^{-3}
M1	1147	-	-	8.24×10^{-4}	2.47×10^{-3}
N-P1	1146	150	23	1.80×10^{-4}	7.07×10^{-4}

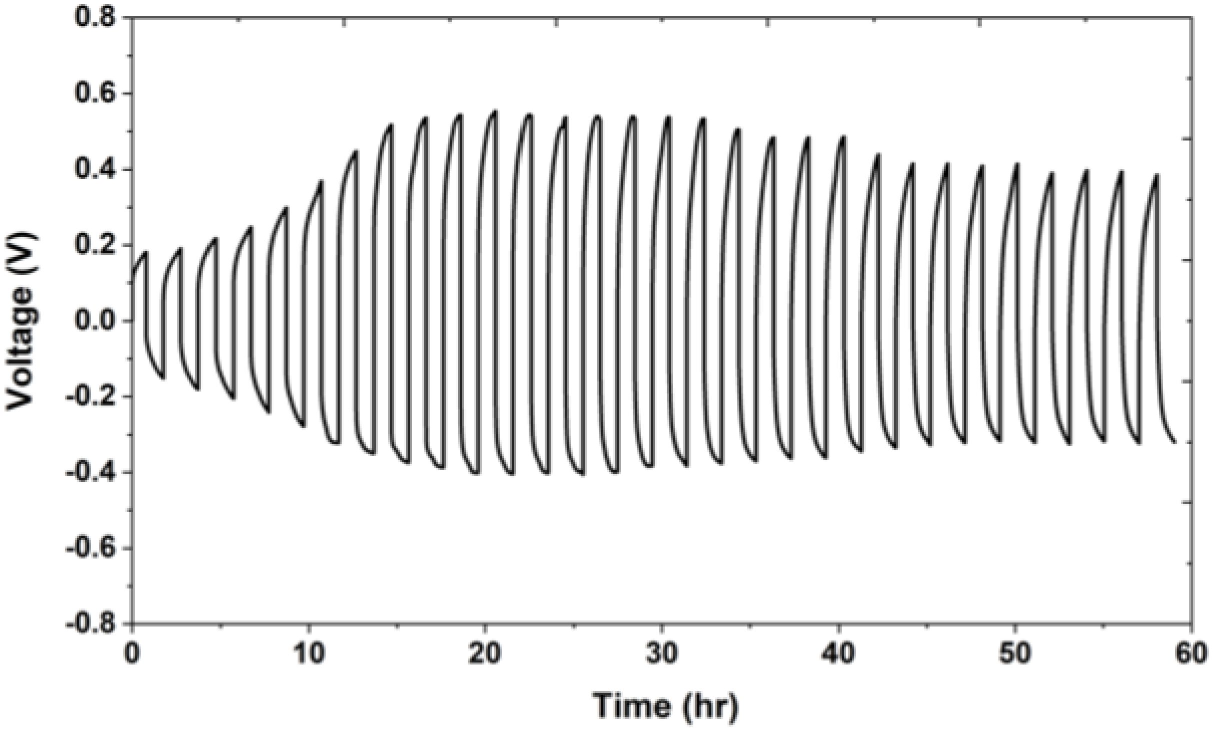

Figure S22. Voltage-time curves of Li|M1 electrolyte|Li symmetric cell at a constant current density of 0.1 mA cm^{-2} over 60 cycles.

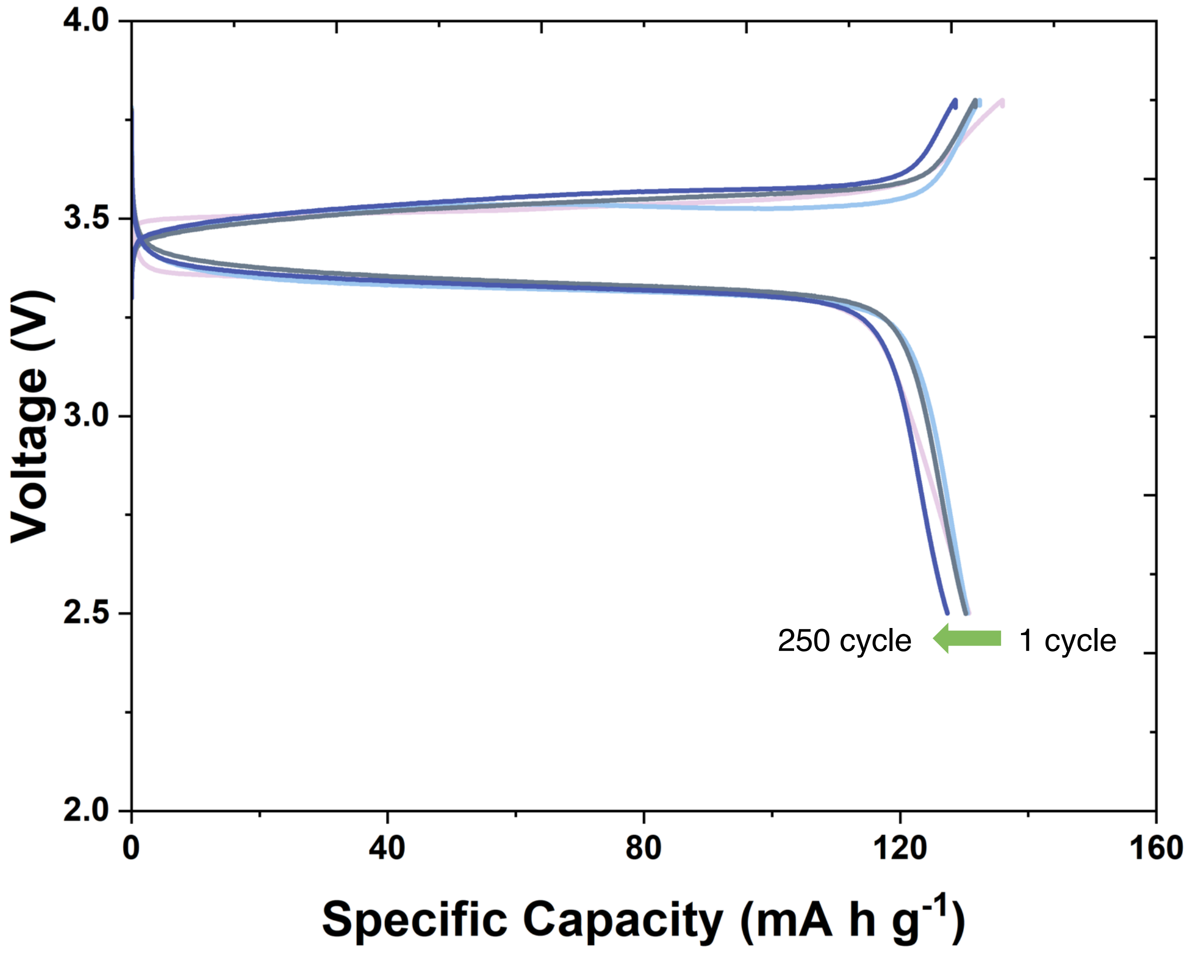

Figure S23. Charge and discharge profiles during cycling experiments of Li|P1 electrolyte|LFP at cycle numbers 1, 50, 100, and 250 cycles at 0.5 C rate.

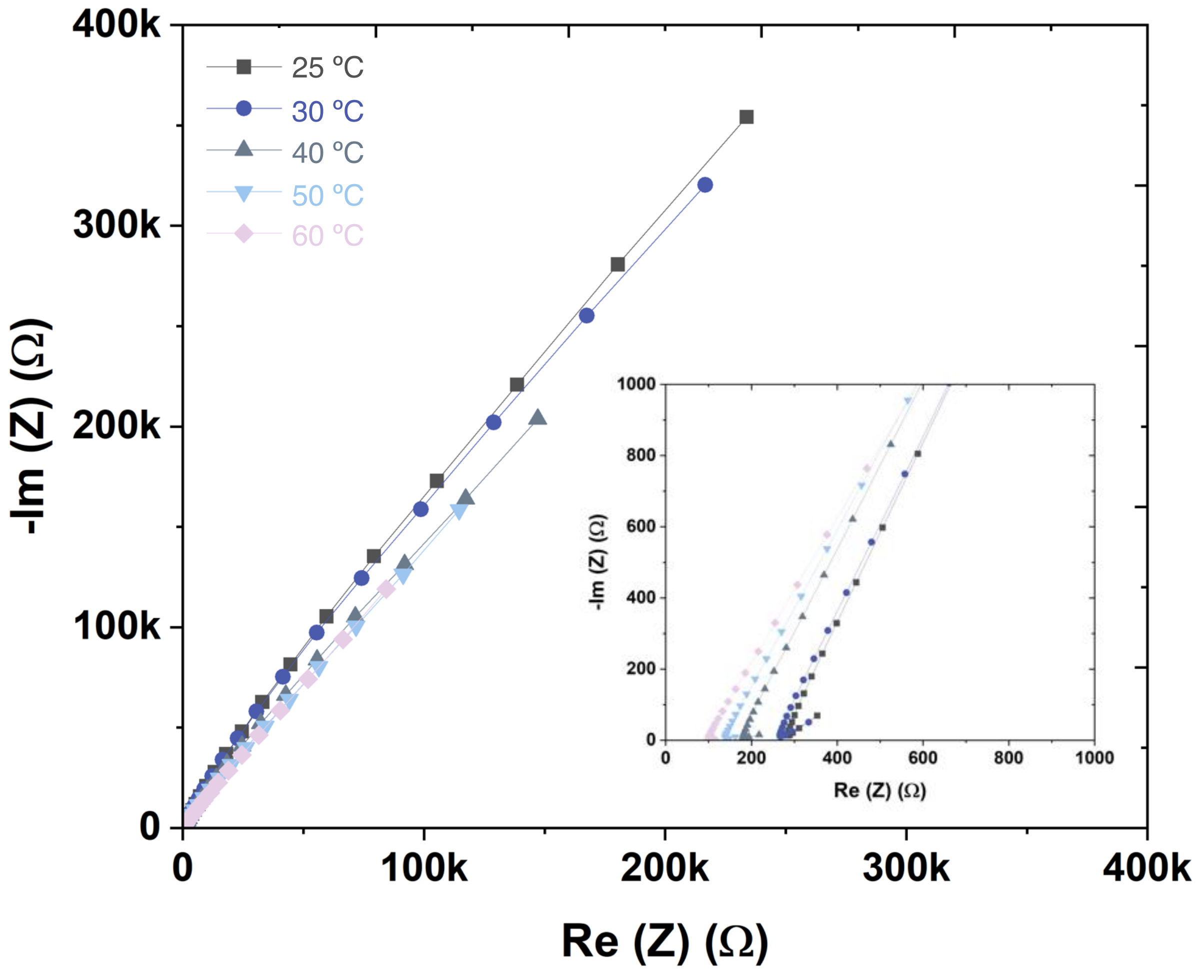

Figure S24. Representative Nyquist plots of polymer electrolytes (P1-4) for ionic conductivity measurements.

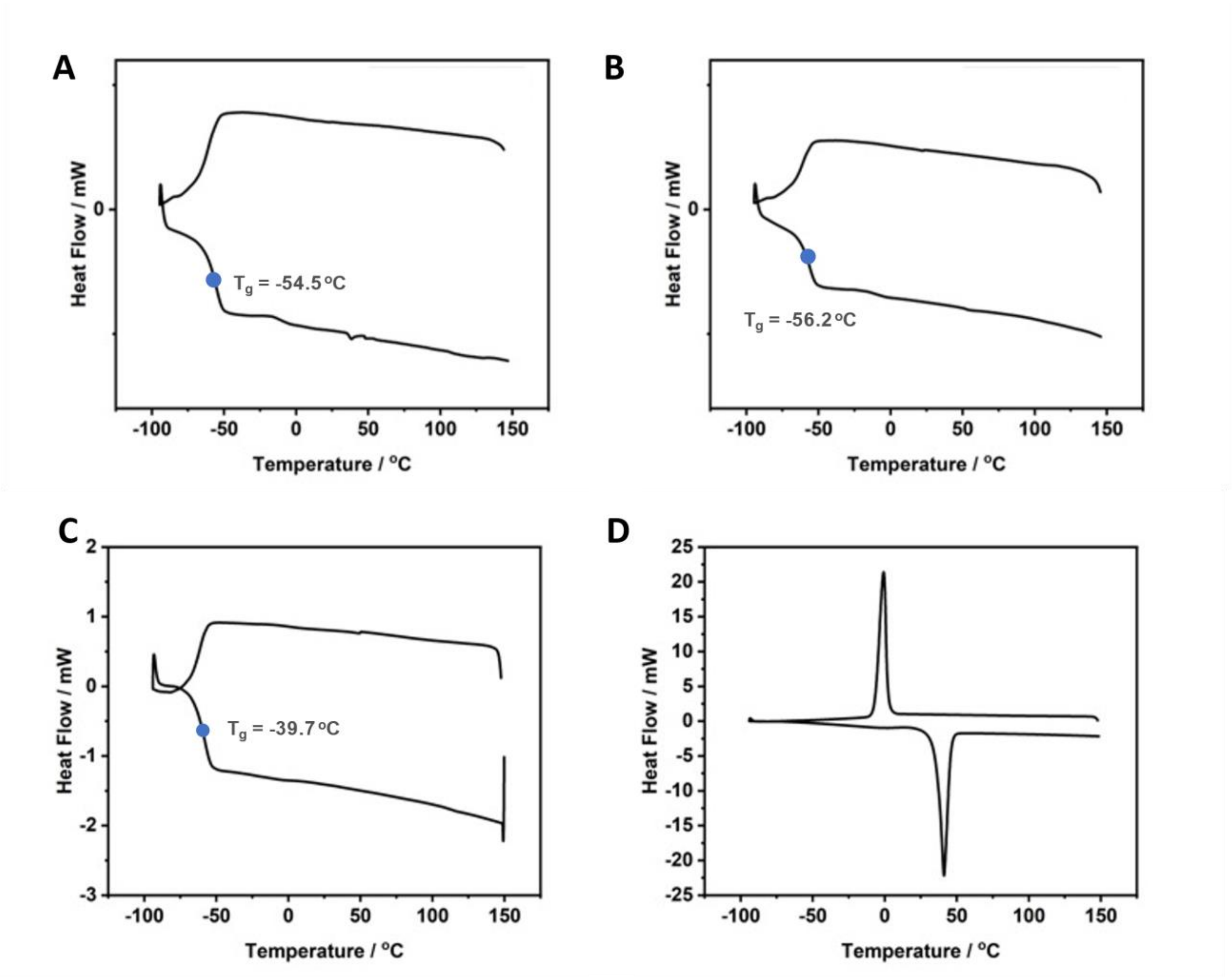

Figure S25. Differential scanning calorimetry of P2-1A (A), P2-1B (B), P2-1C(C), and P2-2A (D).

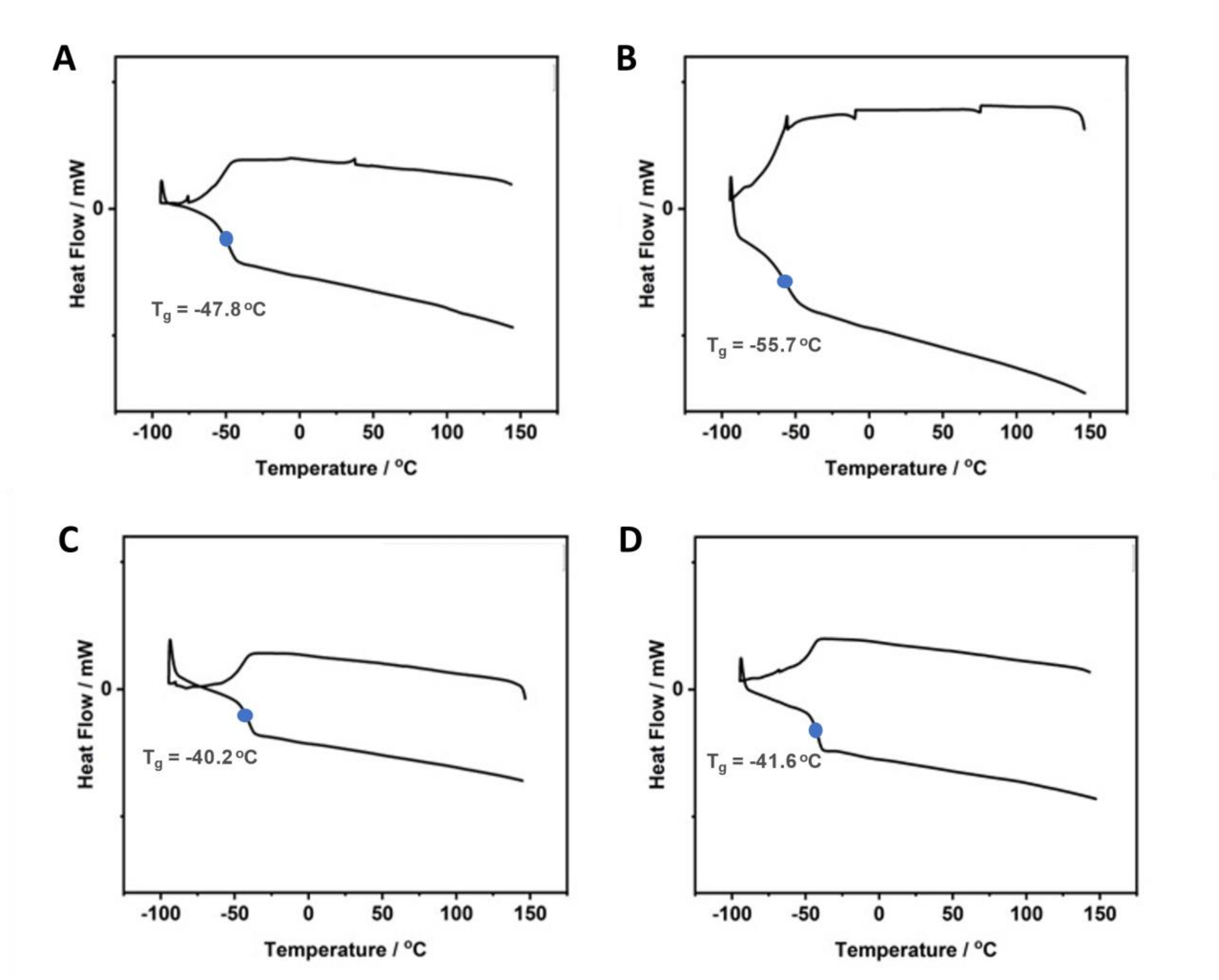

Figure S26. Differential scanning calorimetry of P1 polymer electrolyte after LiTFSI addition: P2-1A (A), P2-1B (B), P2-1C(C), and P2-2A (D).

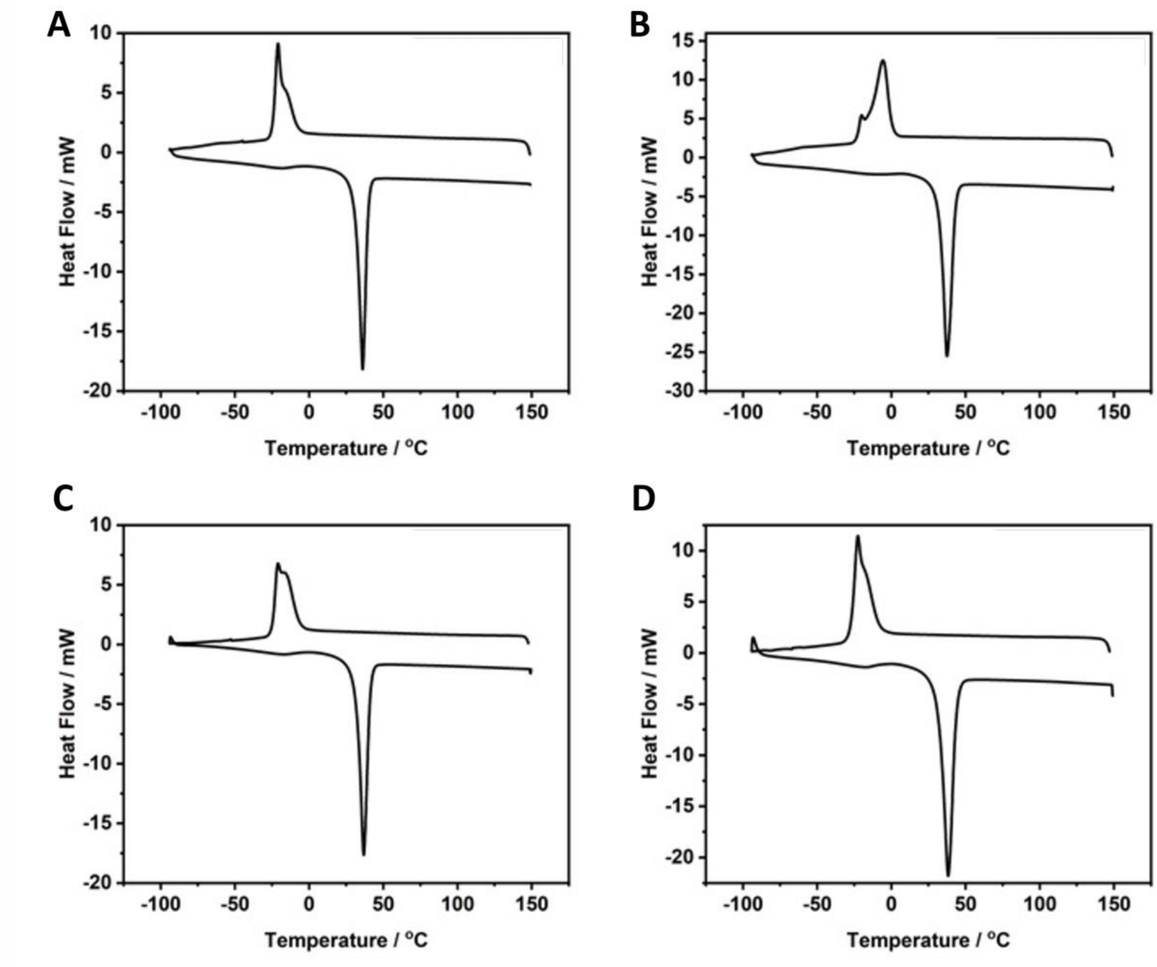

Figure S27. Differential scanning calorimetry of P1-1 (A), P1-2 (B), P1-3(C), and P1-4 (D).

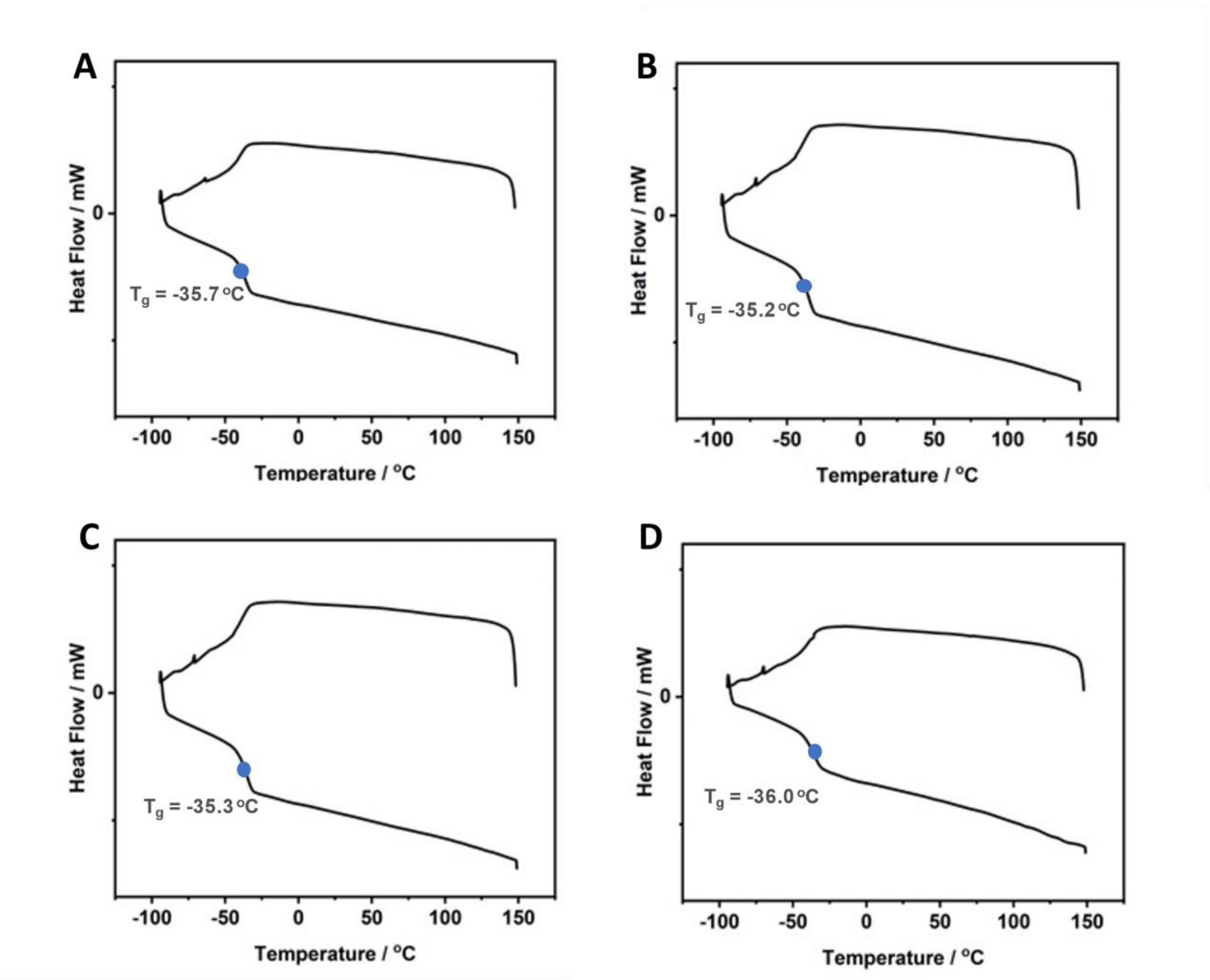

Figure S28. Differential scanning calorimetry of P1 polymer electrolyte after LiTFSI addition: P1-1 (A), P1-2 (B), P1-3(C), and P1-4 (D).

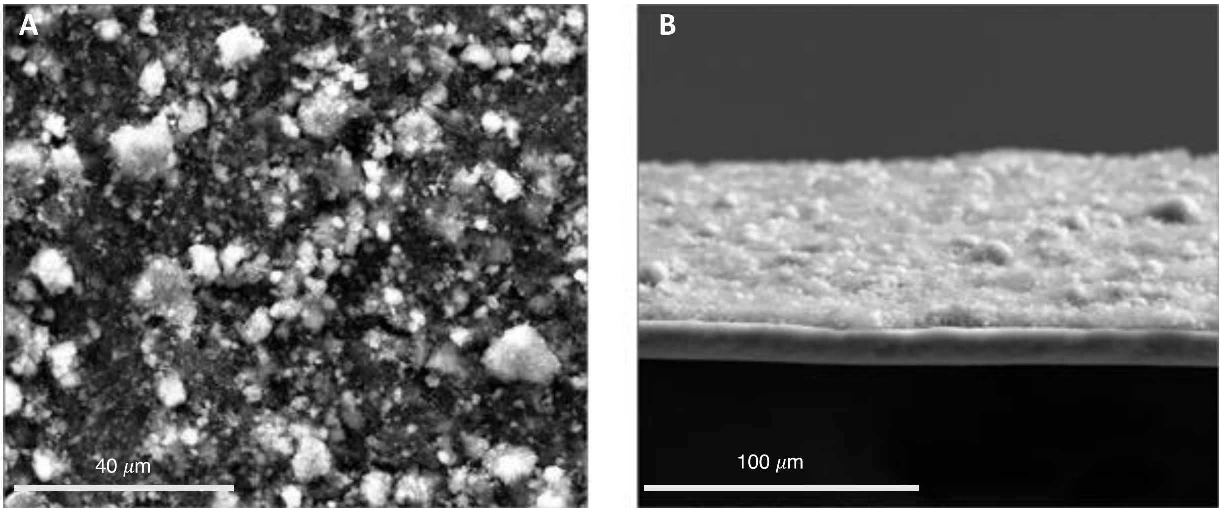

Figure S29. (A) Top-down and (B) cross-sectional scanning electron microscope (SEM) images of LFP film.

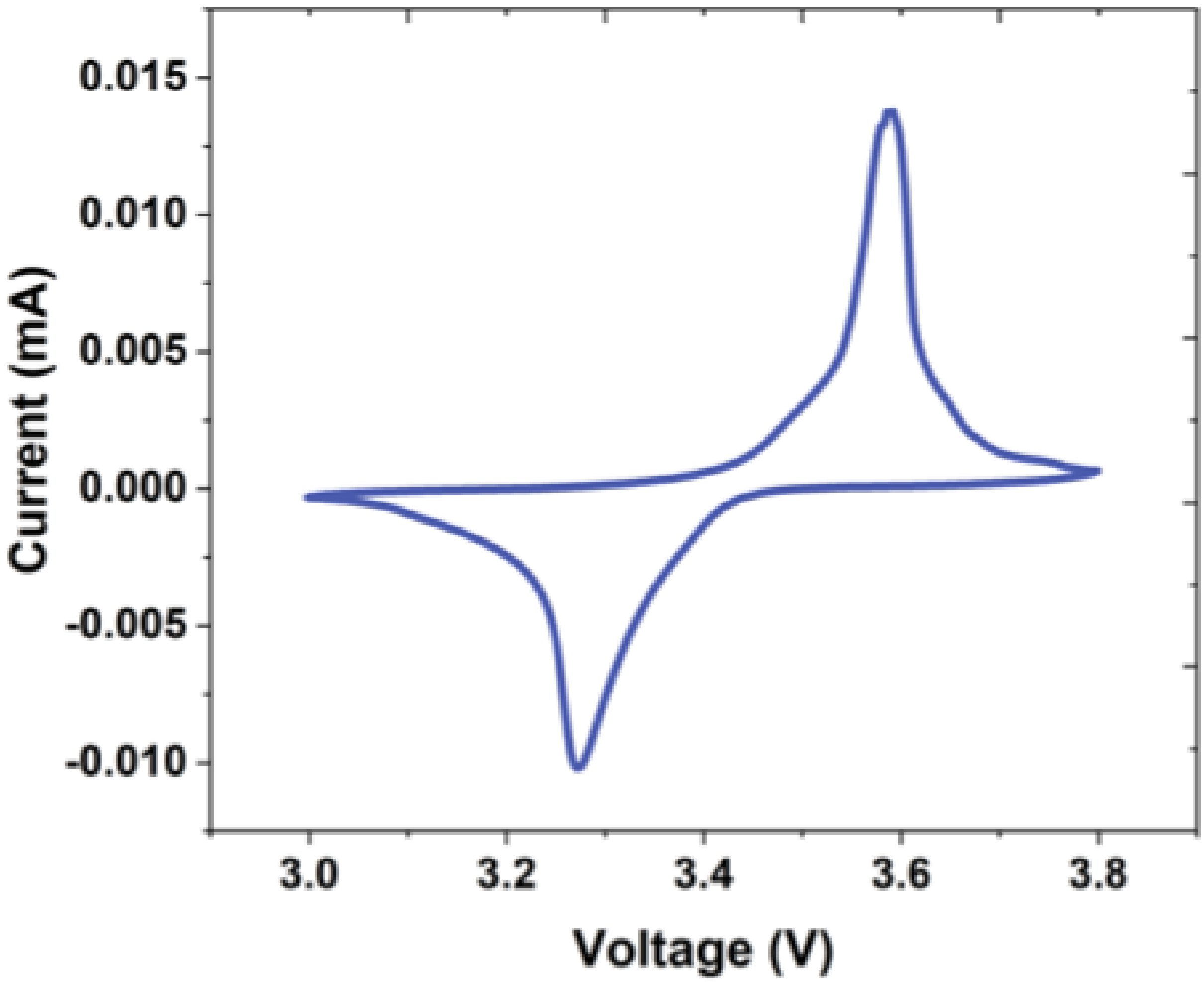

Figure S30. Cyclic voltammetry of Li|P1 electrolyte|LFP at 0.01 mV s^{-1} scan rate

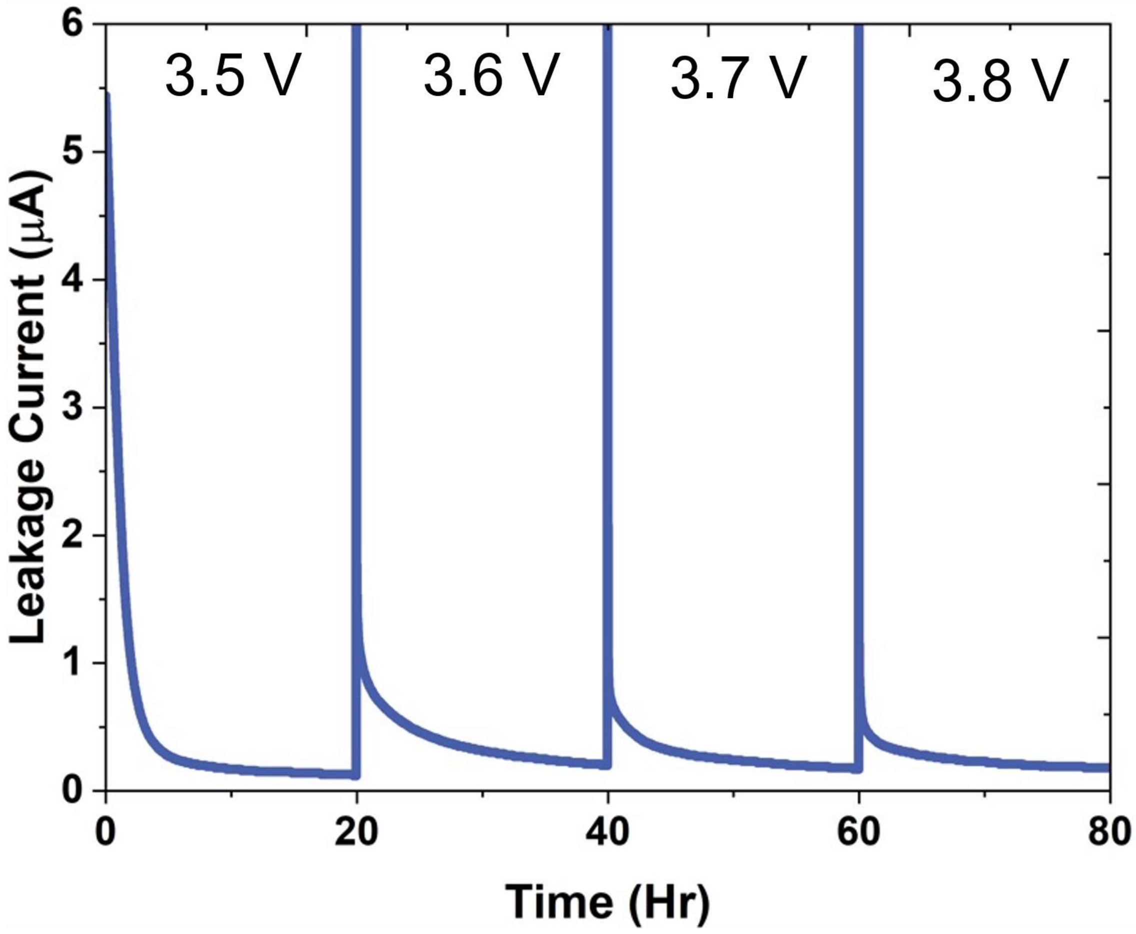

Figure S31. Electrochemical stability of P1 paired with LFP cathode.

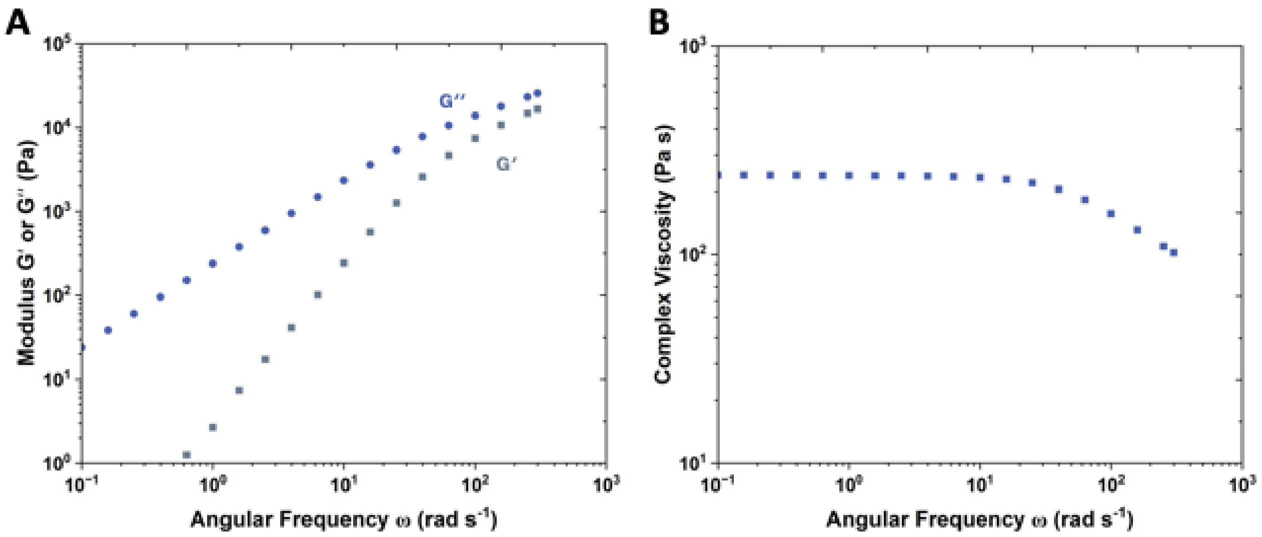

Figure S32. Frequency sweep test of P1 SPE under the constant value of strain amplitude 10% at 25 ^{o}C(A) Storage modulus and loss modulus as a function of angular frequency (B) Complex viscosity as a function of angular frequency.

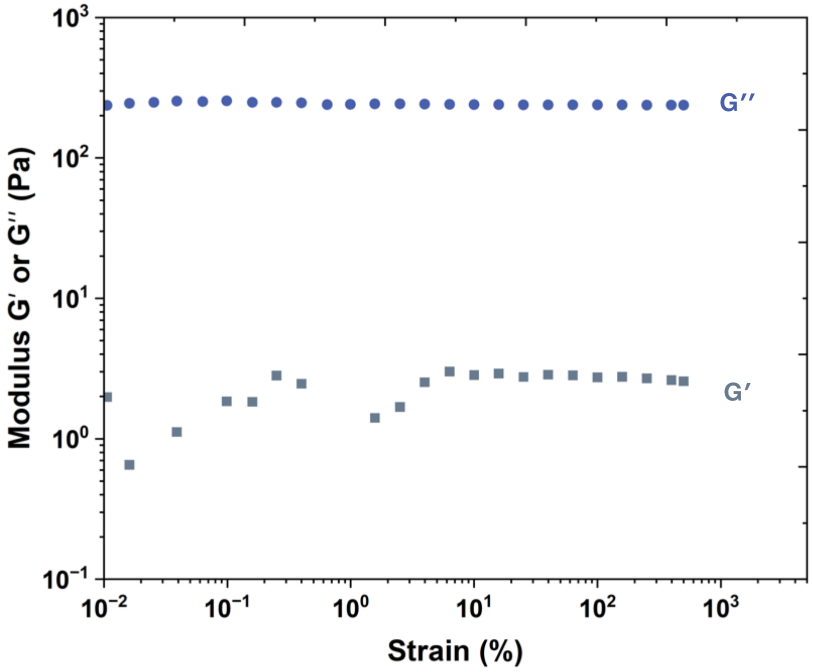

Figure S33. Amplitude sweep test of P1 SPE with an angular frequency of 1 rad s^{-1} . Storage modulus and loss modulus as a function of strain.

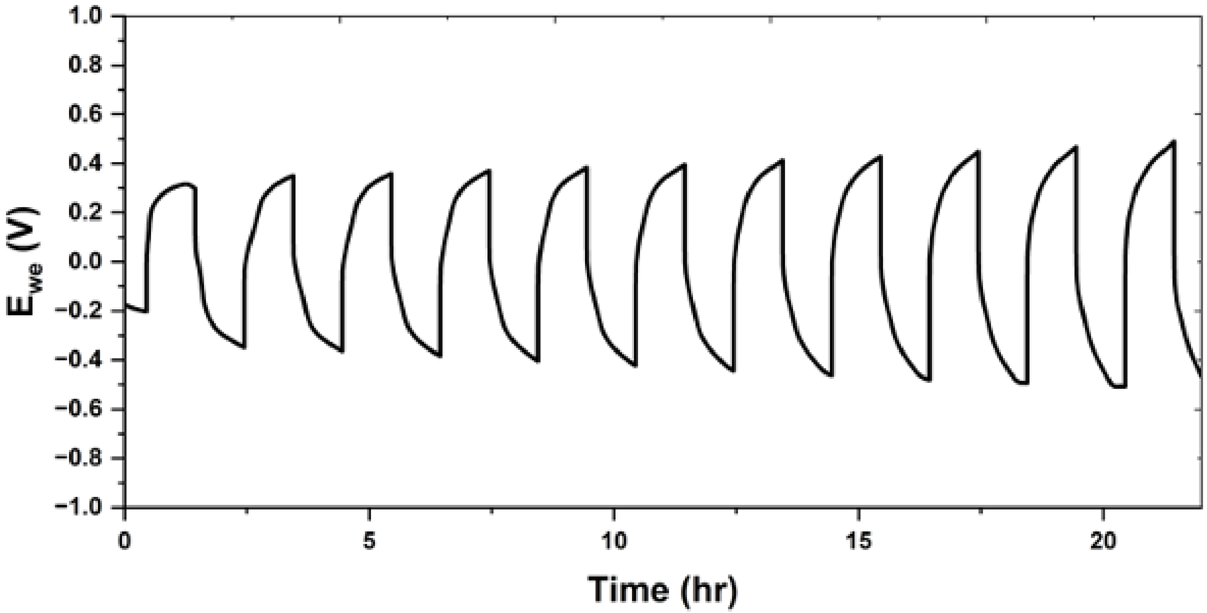

Figure S 34. Galvanostatic cycling of Li|P1 electrolyte|Li symmetric cell at a constant current density of 1 mA cm^{-2} ; 1 mA h cm^{-2} over 11 cycles hours at 50 ^{o}C.

Table S3. Comparison of the polyoxanorbornene (P1) polymer electrolyte to other polymer electrolyte systems.

#	Samples	Structure	σ (S cm^{-1})	References
-	P1-3	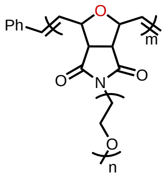	7.12 x 10^{-4} at 25 ^{o}C	This work
-	P2-2A	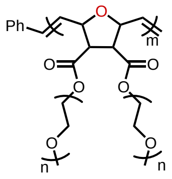	2.93 x 10^{-4} at 25 ^{o}C	This work
-	N-P1	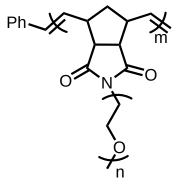 n= 23	1.80 x 10^{-4} at 25 ^{o}C	This work
1	PNG-PTG-PNG	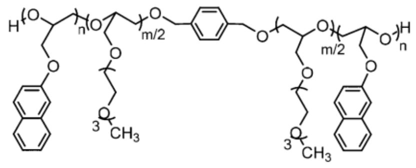	9.62 x 10^{-5} at 30 ^{o}C	[1]
2	PPMALi^{+}-*g*-PEO	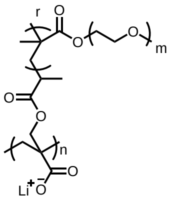	8.87 x 10^{-5} at 30 ^{o}C	[2]
3	Cellulose-based polymer electrolyte	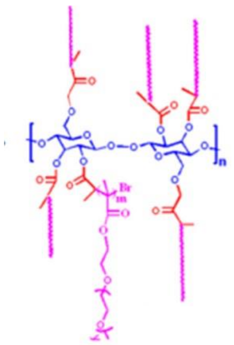	8.09 x 10^{-5} at 30 ^{o}C	[3]

4	Poly(Nb)$_m$-g-PEGME$_{2k}$	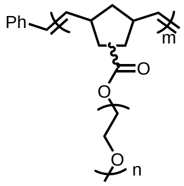	7.34 x 10^{-5} at 25 ^{o}C	[4]
5	PEGDMA/ PEGDA/ PETMP	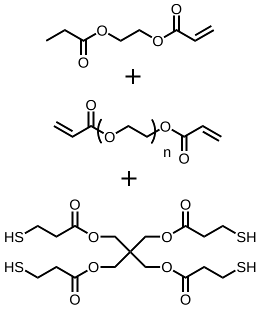	5.76 x 10^{-5} at 30 ^{o}C	[5]
6	PBE 750	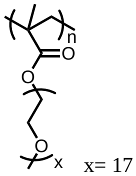	4.87 x 10^{-5} at 25 ^{o}C	[6]
7	*g*Ps-*g*PEO-*g*PS	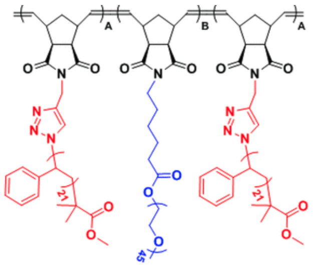	2.26 x 10^{-5} at 25 ^{o}C	[7]
8	S(EO)$_3$	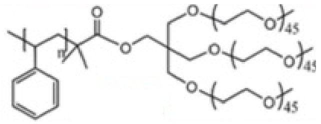	1.09 x 10^{-5} at 25 ^{o}C	[8]
9	*hb*PPEOMA-*s*-PS$_n$ star polymer	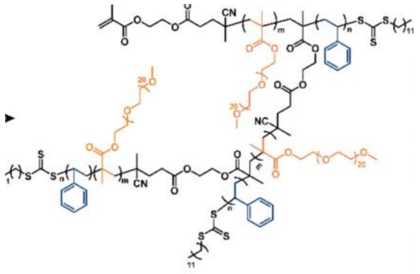	1.10 x 10^{-5} at 25 ^{o}C	[9]
10	P(PO/EM)	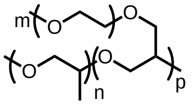	1.16 x 10^{-5} at 28 ^{o}C	[10]

11	PBE 350	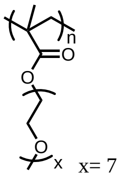	8.06 x 10^{-6} at 25 ^{o}C	[6]
12	PBE 2000	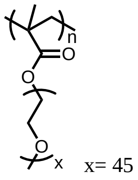	5.62 x 10^{-6} at 25 ^{o}C	[6]
13	DGBE-PEODA$_{600}$	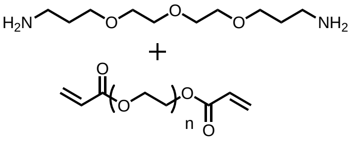	5.02 x 10^{-6} at 30 ^{o}C	[11]
14	PETE-1	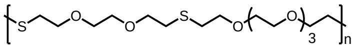	3.09 x 10^{-6} at 27 ^{o}C	[12]
15	PEOMA-TFSI^{-} Li^{+}	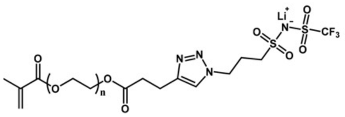	1.54 x 10^{-6} at 25 ^{o}C	[13]
16	Poly(7-oxanorbornene) with pendant oligoethyleneoxy-functionalized	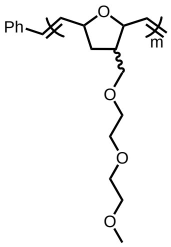	1.27 x 10^{-6} at 25 ^{o}C	[14]

$$\sigma = \frac{L}{R \times S}$$ (Equation S1)

Where *L* is the thickness of polymer electrolytes, *A* is the area of the electrolyte, and *R* is the impedance determined from the Nyquist plot.

References

[1] B. Kim, C.-G. Chae, Y. Satoh, T. Isono, M.-K. Ahn, C.-M. Min, J.-H. Hong, C. F. Ramirez, T. Satoh, J.-S. Lee, *Macromolecules* **2018**, *51*, 2293.

[2] S. Li, K. Jiang, J. Wang, C. Zuo, Y. H. Jo, D. He, X. Xie, Z. Xue, *Macromolecules* **2019**, *52*, 7234.

[3] S. Wang, L. Zhang, Q. Zeng, X. Liu, W.-Y. Lai, L. Zhang, *ACS Sustainable Chem. Eng.* **2020**, *8*, 3200.

[4] D. Rosenbach, N. Mödl, M. Hahn, J. Petry, M. A. Danzer, M. Thelakkat, *ACS Appl. Energy Mater.* **2019**, *2*, 3373.

[5] Z. Wei, S. Chen, J. Wang, Z. Wang, Z. Zhang, X. Yao, Y. Deng, X. Xu, *J. Mater. Chem. A* **2018**, *6*, 13438.

[6] X. Ji, L.-L. Xiao, Y. Zhang, K. Yue, X. Zhou, Z.-H. Guo, *ACS Appl. Energy Mater.* **2022**, *5*, 8410.

[7] C. M. Bates, A. B. Chang, N. Momčilović, S. C. Jones, R. H. Grubbs, "ABA Triblock Brush Polymers: Synthesis, Self-Assembly, Conductivity, and Rheological Properties," DOI 10.1021/acs.macromol.5b00880can be found under https://pubs.acs.org/doi/full/10.1021/acs.macromol.5b00880, **2015**.

[8] D. Lee, H. Y. Jung, M. J. Park, *ACS Macro Lett.* **2018**, *7*, 1046.

[9] Y. Chen, Y. Shi, Y. Liang, H. Dong, F. Hao, A. Wang, Y. Zhu, X. Cui, Y. Yao, *ACS Appl. Energy Mater.* **2019**, *2*, 1608.

[10] Q. Wang, Z. Cui, Q. Zhou, X. Shangguan, X. Du, S. Dong, L. Qiao, S. Huang, X. Liu, K. Tang, X. Zhou, G. Cui, *Energy Storage Materials* **2020**, *25*, 756.

[11] J. Wang, C. Zhang, Y. Zhang, G. Chen, R. Poli, X. Xie, Z. Xue, *Macromolecules* **2023**, DOI 10.1021/acs.macromol.2c02230.

[12] J. M. Sarapas, G. N. Tew, *Macromolecules* **2016**, *49*, 1154.

[13] S. Li, A. I. Mohamed, V. Pande, H. Wang, J. Cuthbert, X. Pan, H. He, Z. Wang, V. Viswanathan, J. F. Whitacre, K. Matyjaszewski, *ACS Energy Lett.* **2018**, *3*, 20.

[14] H. R. Allcock, J. D. Bender, R. V. Morford, E. B. Berda, *Macromolecules* **2003**, *36*, 3563.
